# Patterns and Clinical Efficacy of Biologics Switching in Patients With Severe Asthma: A Systematic Review and Meta‐Analysis

**DOI:** 10.1111/all.70333

**Published:** 2026-04-04

**Authors:** Yang Zheng, Ya‐chun Li, Sheng‐jie Li, Meng Xu, Jia‐qian Hu, Wen‐qu Tian, Shi‐wei Chen, Xue‐hui Li, Ying He, Gan Lu, Mübeccel Akdis, Ioana Agache, Cezmi A. Akdis, Ya‐dong Gao

**Affiliations:** ^1^ Department of Allergy The First Affiliated Hospital, Zhejiang University School of Medicine Hangzhou China; ^2^ Yuanshan Public Health Service Center Shenzhen Guangdong China; ^3^ Swiss Institute of Allergy and Asthma University Zurich Davos Switzerland; ^4^ Faculty of Medicine Transylvania University Brasov Romania

**Keywords:** biologics switch, clinical remission, efficacy, severe asthma, systematic review and meta‐analysis

## Abstract

**Background:**

This systematic review (SR) aims to delineate the patterns and rationales for biologic switching in patients with severe asthma and evaluate its efficacy across the clinical remission criteria.

**Methods:**

The SR followed the PRISMA guidelines (PROSPERO CRD420251155819), with searches up to September 2025. Studies reporting on switching of biologics, including anti‐IgE, anti‐IL‐4R/13R, anti‐IL5/5R, and anti‐TSLP, were included. Standardized mean difference (SMD) or mean difference (MD), and pooled relative risk (RR) were calculated for pre‐ and post‐ switch comparisons.

**Results:**

The SR included 49 studies (2292 switched severe asthma patients). The most common switching patterns were mepolizumab‐benralizumab (*n* = 637) and omalizumab‐mepolizumab/benralizumab (*n* = 386 or 305, respectively). Additional switching patterns included transitions from other biologicals to dupilumab or tezepelumab. Suboptimal asthma control (*n* = 1005, 77.0%) was the predominant reason for switching. The switch led to a significant reduction in exacerbations (SMD –1.03, 95% CI: −1.26 to −0.80, *I* ^2^ = 89%), emergency department visits, hospitalizations, and maintenance oral corticosteroid dose and to improved asthma control ACT MD 5.18 (95% CI 4.32 to 6.04, *I* ^2^ = 80%), ACQ MD –1.05 (95% CI –1.26 to −0.83, *I* ^2^ = 45%) and lung function FEV1 MD 0.18 L (95% CI: 0.11 to 0.25, *I* ^2^ = 0%). T2‐biomarkers (blood eosinophils, total serum IgE, FeNO) significantly decreased.

**Conclusion:**

Biologics switching represents a promising strategy supported by high‐quality evidence of its clinical efficacy. Switching should consider clinical remission goals, co‐morbidities, side effects, costs and reimbursement policies, and patient preferences.

## Graphical abstract

This meta‐analysis included 2292 patients with severe asthma who underwent biologic switching across 23 countries/regions. The most prevalent switching pattern was from mepolizumab to benralizumab, with suboptimal asthma control being the primary reason. Biologic switching is probably a promising strategy for achieving clinical remission and improving T2 biomarkers.

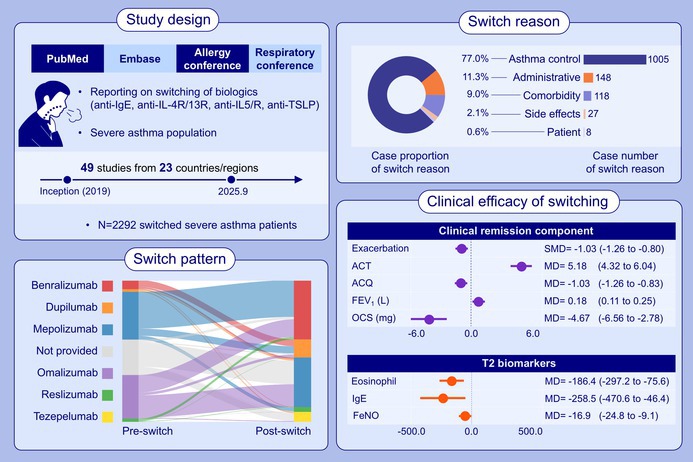

**Abbreviations**
ACQ: asthma control questionnaire
ACT: asthma control test
AQLQ: asthma quality of life questionnaire
FeNO: fractional exhaled nitric oxide
FEV1: forced expiratory volume in one second
IgE: immunoglobin E
IL: interleukin
mOCS: maintenance oral corticosteroid
QoL: quality of life
TSLP: thymic stromal lymphopoietin

## 1 Introduction

Asthma is a common chronic airway inflammatory disease that affects more than 300 millions of people worldwide, imposing a persistent and heavy burden on individuals' quality of life (QoL) and healthcare systems [1]. Severe asthma accounts for only 5%–10% of the total asthma population, but it places a disproportionately huge burden on healthcare system resources, thus being a major challenge [2]. In recent years, with the improved understanding of the core pathological mechanism of type 2 (T2) inflammation, a series of targeted biologics have been developed and implemented into clinical practice, which has greatly revolutionized the treatment of severe asthma [3, 4].

With the emergence of biological drugs, the “clinical remission” of asthma has been introduced as a superior treatment goal. This concept holds that a genuine clinical therapeutic effect should simultaneously reach the following four key indicators: no exacerbations, normalization of type 2 (T2) biomarkers, discontinuation of maintenance oral corticosteroids (mOCS), and stable or improved lung function [5]. Within this framework, a large number of studies have begun to focus on evaluating the clinical remission rate achieved through biologics therapy [6, 7]. However, real‐world data showed that a considerable proportion of patients still have a poor response to the initial biological treatment [8]. A meta‐analysis of observational studies on the real‐world effectiveness of omalizumab in severe allergic asthma showed a responder rate of 65.2%, 68.1%, and 71.1% at week 16, week 32, and week 48, respectively, with the remaining patients having a poor or no response to treatment [9]. Another real‐world study on dupilumab showed that only 30% of patients attained clinical remission [10]. With the emergence of new biologicals, switching from one biological agent to another with similar or different mechanisms has become a therapeutic strategy for non‐responders [11]. Preliminary evidence indicates that switching biologics in patients with severe asthma may yield favorable clinical outcomes. In one study, 145 patients previously treated with omalizumab but experiencing more than two exacerbations annually were switched to mepolizumab, resulting in significant improvements in asthma control, exacerbation rates, lung function, and blood eosinophil counts [12]. Similarly, a multicenter retrospective study in Japan involving 27 severe asthma patients switched from omalizumab, mepolizumab, or benralizumab to dupilumab demonstrated significant enhancements in lung function and ACT scores [13].

Although the aforementioned studies support the switching of biological agents in severe asthma, the evidence base in this field remains limited. Previous reports were primarily derived from small‐scale observational studies with heterogeneous designs, lacking standardized switching algorithms. Furthermore, patterns and rationale for switching are not well established, and its efficacy is usually incompletely assessed using limited or non‐quantitative outcomes [14]. Currently, there is still a lack of comprehensive and integrated evaluation that fulfills the multiple dimensional criteria of “clinical remission”.

To address these gaps, this study aims to delineate the patterns and rationales for biologic switching in patients with severe asthma by systematically evaluating the evidence from both observational studies and randomized clinical trials. Additionally, the meta‐analysis quantitatively evaluated the efficacy of biologic switching across all components of clinical remission criteria: exacerbations, asthma control, asthma‐related QoL, lung function, mOCS dose, and T2‐biomarkers.

## 2 Methods

This systematic review (SR) and meta‐analysis was conducted systematically under the Preferred Reporting Items for Systematic Review and Meta‐Analyses (PRISMA) guidelines. The protocol was registered in PROSPERO (CRD420251155819).

### 2.1 Search Strategy

The SR and meta‐analysis included data from PubMed/MEDLINE, EMBASE, and allergy or respiratory‐related conference database, including EAACI (European Academy of Allergy and Clinical Immunology Annual) Congress, AAAAI (American Academy of Allergy, Asthma and Immunology) Congress, World Allergy Congress, European Respiratory Society (ERS) Congress, American Thoracic Society (ATS) International Congress, Chest Annual Meeting, etc. A combination of MeSH and free‐text terms was used. The sources were searched from their inception to September 30, 2025. We also hand‐searched the bibliographies of relevant systematic reviews to identify additional qualifying papers. Search terms included: (“mepolizumab” OR “benralizumab” OR “omalizumab” OR “dupilumab” OR “tezepelumab” OR “anti‐IL‐4” OR “anti‐IL‐5” OR “anti‐IL‐13” OR “anti‐IgE” OR “anti‐TSLP”) AND “severe asthma” (AND “biologic switch” OR “antibody switch”). Details on the methodology and content of searches are provided in Data S1. No language restriction was imposed for the search. Articles and conference records were managed in EndNote X9 (Clarivate Analytics, Philadelphia, USA).

### 2.2 Inclusion and Exclusion Criteria

Inclusion criteria were: (1) cohort studies or randomized clinical trials involving severe asthma population. (2) Original article, research letter, conference abstract (need full‐text examination). (3) Studies reporting on switching of biologics including but not limited to anti‐IgE, anti‐IL‐4R/13R, anti‐IL5/R, anti‐TSLP. (4) studies reporting on clinical remission parameters: exacerbations, emergency department visit, hospitalization, mOCS use, asthma control (asthma control test (ACT) and/or asthma control questionnaire (ACQ)), pulmonary function test (pre‐bronchodilator forced expiratory volume in 1 s (FEV1)), blood eosinophil counts, total serum immunoglobin E (IgE), fractional exhaled nitric oxide (FeNO), asthma quality of life (AQLQ).

Studies were excluded if they were: (1) case reports or case series reviews. (2) contained duplicated data. (3) lacked original data or had non‐extractable data or did not report on prespecified outcomes.

### 2.3 Data Extraction

Two authors (Y.Z., Y.L.) independently screened the titles and abstracts of all articles to exclude those reporting on studies not fulfilling the prespecified inclusion criteria. Full‐text articles were then evaluated, cross‐checked for validity and accuracy. Disagreements were resolved through consensus by all authors. Data from eligible studies were extracted using a standardized template in Microsoft Excel 2016 (Microsoft, Washington DC, USA), encompassing: (1) basic study characteristics (title, first author, journal, publication year, study period, region); (2) epidemiologic data (publication type, study design, sample size, characteristics of included population); (3) clinical description of biologics use experience (prior‐switch biologic type, post‐switch biologic type, switch reasons); (4) prior‐ and post‐switched clinical remission outcomes, described as mean (standard deviation) or median (interquartile range) of exacerbation rate, emergency department visit rate, hospitalization rate, maintenance OCS dose, FEV1, ACT, ACQ, AQLQ, blood eosinophil counts, total serum IgE, FeNO; for studies that did not directly report rates, the number of exacerbations, emergency department visits, and hospitalizations were first extracted and converted to rates prior to synthesis.

### 2.4 Risk of Bias and Quality Assessment

The risk of bias and quality of included studies was assessed using the Newcastle‐Ottawa Scale (NOS) for cohort studies and the Cochrane risk‐of‐bias tool for randomized trials (RoB 2) [15, 16]. The NOS assessed three domains: selection, comparability, and outcome. The sum of the points represented the overall bias risk of each study, with 7–9 points defined as high quality for cohort and case–control studies and 8–10 points defined as high quality for cross‐sectional studies [17, 18]. RoB 2 has five bias domains: randomization process, deviations from intended interventions, missing outcome data, measurement of the outcome, and selection of the reported result. An overall risk of bias is judged based on performance in each domain with “low risk of bias”, “some concerns”, or “high risk of bias” [16]. Egger's regression‐based test and visual inspection of the funnel plot were performed to assess publication bias in meta‐analyses with a large number of included studies (≥ 10), while a file drawer analysis (fail‐safe *N* calculation) was used to evaluate publication bias in meta‐analyses with fewer studies (< 10) [19]. Small study effects were considered for each meta‐analysis that included at least ten studies, through visual inspection of funnel plots and the application of Egger's tests [20]. Leave‐one‐out sensitivity analysis was conducted to assess the robustness of the final results. As 14 conference abstracts were included for analysis, a post hoc sensitivity analysis of publication type was conducted by excluding the data extracted from conference abstracts.

### 2.5 Statistical Analysis

The study conducted a meta‐analysis incorporating both continuous and binary severe asthma‐related outcome measures. For studies that reported multiple independent outcomes, all relevant data were extracted. When a study reported outcomes at multiple timepoints, we chose the one closest to 1 year duration of treatment. Continuous outcomes using similar scales are reported as mean differences (MD) with 95% CI between pre‐ and post‐biologic switches, continuous data with different scales are reported as standardized mean difference (SMD) with 95% CI using the Hedge g method. For binary outcomes, pooled effect sizes are reported as relative risk (RRs) with 95% CI using the DerSimonian and Laird random‐effects models between pre‐ and post‐biologic switches. For rates estimation, pooled rates were conducted with Freeman‐Tukey double arcsine transformation of the original rate to stabilize the variance and to reduce the effect of extreme values [21]. For studies that reported only medians and interquartile ranges (IQRs), the means and standard deviations (SDs) were estimated using the methods described by Luo et al. [22]. For missing data, a single imputation method that borrows information from the observed outcomes was used. Specifically, the missing values of standard deviation were imputed multiplying the reported mean by the mean or median coefficient of variation calculated from all studies with complete data [23]. Heterogeneity was assesed using *I* ^2^ and by conducting a subgroup analysis by the types of post‐switch biologics. Data were analyzed using R version 4.3.1 (R Core Team, Vienna, Austria). A *p* value < 0.05 was considered statistically significant.

## 3 Results

### 3.1 Description of Studies Included

**FIGURE 1:**
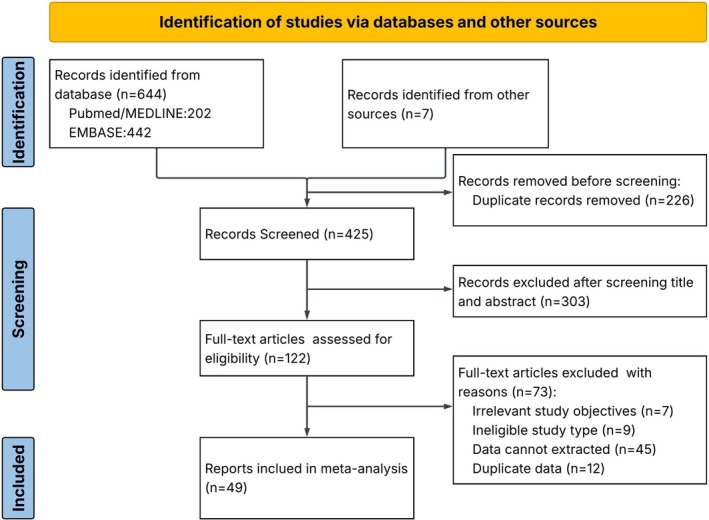
Flow chart of studies selection process in systematic review and meta‐analysis.

The SR screened 651 studies from databases and other sources, and removed 226 duplicated records, with 425 unique records evaluated. The analysis excluded 303 studies that did not meet the inclusion criteria by title or abstract screening, with 122 full‐text articles being independently reviewed. After full‐text review, the SR included 49 reports from 23 countries/regions (Figure 1). Published years range from 2019 to 2025. The analysis included 32 full text articles, 3 research letters, and 14 conference abstracts. Sample size reported in the studies ranged from 4 to 357 participants. The dominant study design was retrospective observational (*n* = 29), 4 prospective observational studies, and one randomized clinical trial (Table 1).

**TABLE 1:** Characteristics of included studies in the systematic review and meta‐analysis.

First author, year	Country/region	Publication type	Study time	Study design	Sample size	Prior‐switch biologics	Post‐switch biologics	Reported clinical outcomes
Akaba, T., 2023	Japan	Article	2009.5–2019.9	Retrospective observational cohort study	20	OMA17, MEP2, DUP1; 8 patients received ≥ 3 biologic switch	MEP 14, DUP 6, BEN 6, OMA4	Clinical exacerbation, OCS dose, ACT
Cakmak, M. E., 2024	Türkiye	Article	2021.1–2023.7	Retrospective observational cohort study	15	OMA 15	MEP	Clinical exacerbation, hospitalization, OCS use, Eos, FEV1, ACT
Cameli, P., 2024	Italy	Article		ANANKE (NCT04272463): Multicenter retrospective observational study	38	OMA 21, MEP 17	BEN	Clinical exacerbation, OCS dose, OCS use, FEV1, ACT
Caminati, M., 2023	Italy	Article	2015.1–2025.12	Multicenter retrospective observational study	30	MEP	BEN	Clinical exacerbation, OCS dose, Eos, FeNO, FEV1, ACT
Carpagnano, G. E., 2020	Italy	Article		Retrospective observational cohort study	41	OMA 41	MEP	Clinical exacerbation, hospitalization, OCS user, Eos, sputum Eos, FeNO, ACT, ACQ‐7, AQLQ, FEV1
Carstens, D., 2023	United States	Article	2017.11–2019.6	Retrospective observational cohort study	349	OMA 205, MEP 144	BEN	Clinical exacerbation, OCS use, OCS dose
Chapman, K. R., 2019	Argentina, Belgium, Canada, France, Germany, Netherlands, Spain, Sweden, United States	Article	2016.3–2017.5	OSMO (Omalizumab Switch to Mepolizumab study): Open‐label, single‐arm, multicenter trial (NCT02654145)	145	OMA	MEP	Clinical exacerbation, ACQ‐5, Eos, FEV1
Cheng, S. L., 2025	Taiwan	Article	2018.11–2020.10	REMIT: Retrospective observational cohort study	42	Not provided	MEP	Clinical exacerbation, ER visit, hospitalization, mOCS dose, Eos, ACT, FEV1
Fernández, A. G. B., 2022	Spain	Article	2020.6–2021.6	Retrospective observational cohort study	40	OMA 16, MEP 24	BEN	Clinical exacerbation, ER visit, Hospitalization, OCS use, ACT, FEV1, FeNO, Eos
Hashimoto, S.,2022	Netherlands	Article		Dutch severe asthma registry (RAPSODI): Multicenter retrospective observational cohort study	78	OMA 3, MEP 66, BEN 8, DUP 1	RES	Clinical exacerbation, ER visit, hospitalization, mOCS dose, OCS us
Higo, H., 2023	Japan	Article	2019.5–2021.9	Multicenter retrospective observational cohort study	27	OMA 3, MEP 3, BEN 21	DUP	FEV1, ACT, FeNO
Jackson, D., 2024	United Kingdom	Article		BPAP study (NCT05932849): Multicenter retrospective observational cohort study	113	MEP 79, RES 17, OMA 14	BEN	Clinical exacerbation, ACQ‐6, AQLQ
Jiménez‐Gómez, M., 2024	Spain	Article		Retrospective observational cohort study	9	RES 3, DUP 2, BEN 2, OMA 2	TSLP	Clinical exacerbation, OCS use, FEV1, ACT
Katran, Z. Y., 2023	Türkiye	Article	2015.1–2022.6	Retrospective observational cohort study	4	OMA 4	MEP	Eos, IgE, FEV1, ACT
Lafarge, L., 2024	Belgium	Article	2006–2023	Monocenter retrospective observational cohort study	36	MEP, BEN, RES, OMA	MEP 9, BEN 20, RES 7	Clinical exacerbation, ACT, OCS dose
Mansur, A. H., 2023	United Kingdom	Article	2016–2020	UK Severe Asthma Registry (UKSAR): Retrospective observational cohort study	109	OMA, BEN, MEP	OMA, BEN, MEP	Clinical exacerbation, mOCS dose, OCS use, Eos, FeNO, FEV1, ACQ‐6
Naumova, V. V., 2023	Russia	Article	2019.6–2020.10	Retrospective observational cohort study	14	OMA 5, BEN 5, MEP 3, RES 1	DUP	OCS use, ACT, FEV1, AQLQ
Pelaia, C., 2021	Italy	Article		Multicenter retrospective observational cohort study	20	OMA	BEN	Clinical exacerbation, hospitalization, OCS dose, OCS use, ACT, FEV1, Eos
Rosso, C., 2024	Italy	Article	2021.12–2023.8	Retrospective observational cohort study	20	OMA, MEP, BEN	DUP	Eos, IgE, ACT
Trevor, J., 2021	United States	Article	2018.2–2020.2	CHRONICLE (NCT03373045): Prospective observational cohort study	62	Between IgE and other than anti‐IgE		Clinical exacerbation, ED visit, hospitalization
Valery, S., 2024	France	Article	2019.9–2022.9	RAMSES cohort: Observational cohort study (Target trial emulation)	151	IL‐5/R: MEP 111, BEN 40	DUP 103, another IL‐5/R 48	Clinical exacerbation, OCS dose, ACT, AQLQ, FEV1
Ais‐Daza, A., 2024	Spain	Conference		Ambispective observational cohort study	21	MEP 3, OMA 11, BEN 5, RES 2	DUP	Clinical exacerbation, ER visit, hospitalizations, OCS use, FEV1, ACT, IgE
Al‐Lehebi, R. O., 2022	Saudi Arabia	Conference	2017.1–2021.8	Retrospective observational cohort study	14	MEP	DUP	Clinical exacerbation, OCS user, ACT, Eos, IgE, FEV1
Altous, M., 2021	Australia	Conference		Retrospective observational cohort study	16	OMA‐BEN 7, MEP‐BEN 7, OMA‐MEP 2		Clinical exacerbation, ACQ‐5, FEV1
Arismendi, E., 2024	Spain	Conference		Multicenter retrospective observational cohort study	58	OMA 36, others (not provided)	BEN	Clinical exacerbation, ACT, FEV1
Bagnasco, D., 2019	Italy	Conference		Retrospective observational cohort study	27	OMA	MEP	Clinical exacerbation, hospitalizations, OCS use, FEV1, ACT
Corbridge, T., 2024	United States	Conference	2021.3–2022.9	Retrospective observational cohort study	89	OMA 35, BEN 28, DUP 26	MEP	Clinical exacerbation, OCS use
D'Amato, M., 2019	Italy	Conference		Retrospective observational cohort study	20	OMA	MEP	Clinical exacerbation, ACT
Deb, A., 2023	United States	Conference	2016.10–2019.3	Retrospective observational cohort study	357	OMA, BEN, RES, DUP	MEP	Clinical exacerbation, OCS use
Gates, J., 2023	United Kingdom	Conference		Retrospective observational cohort study	32	BEN 23, MEP 13	DUP	Clinical exacerbation, mOCS dose, ACQ‐6, AQLQ, FEV1
Jackson, D. J., 2024	United Kingdom	Conference	2019.5–2021.10	BPAP study: Retrospective observational cohort study	92	MEP	BEN	Clinical exacerbation, OCS use, mOCS dose, ACQ‐6, AQLQ
Naderisemiromi, M., 2023	United Kingdom	Conference		Retrospective observational cohort study	58	MEP	BEN	Clinical exacerbation, OCS dose, Eos, sputum Eos, ACQ‐6, AQLQ
Nopsopon, T., 2024	United States	Conference		Retrospective observational cohort study	46	Not provided	TSLP	Clinical exacerbation
Okawa, K., 2022	Japan	Conference	2016–2021	Retrospective observational cohort study	9	MEP to BEN 6; BEN to MEP 3		ACT
Tavernier, G., 2021	United Kingdom	Conference		Prospective observational cohort study	16	MEP	BEN	Sputum Eos, Eos, ACQ, AQLQ, OCS dose
Kallieri, M., 2024	Greece	Letter		RELIght: Multicenter prospective observational cohort study (post hoc analysis)	60	OMA	MEP	Clinical exacerbation, OCS dose, Eos, ACT, FEV1
O'reilly, E., 2022	Ireland	Letter	2018.9–2020.9	Retrospective observational cohort study	10	OMA	BEN 6, MEP 4	Clinical exacerbation, hospitalization, OCS dose, Eos, FEV1, ACQ
Drick, N., 2020	Germany	Article	until 2019.05	Multicenter retrospective observational cohort study	60	MEP 48, RES 12	BEN	Clinical exacerbation, OCS use, OCS dose, ACT, FEV1, FeNO, Eos, IgE
Kavanagh, J. E., 2021	United Kingdom	Letter		Retrospective observational case‐series study	33	MEP	BEN	Clinical exacerbation, OCS use, OCS dose, FEV1, Eos, FeNO, ACQ‐6, miniAQLQ
Martinez‐Moragon, E., 2021	Spain	Article	2018.3–2018.12	ORBE study: Multicenter retrospective observational cohort study	27	MEP 24, RES 3	BEN	Clinical exacerbation, ED visit, hospitalization, OCS use, OCS dose, FEV1, Eos, ACT, miniAQLQ
Mummler, C., 2021	Germany	Article		Retrospective observational cohort study	38	MEP 11, RES 2, BEN 9, OMA 6	DUP	Clinical exacerbation, OCS dose, ACT, Eos, FeNO, IgE
Numata, T., 2020	Japan	Article	2018.7–2019.12	Retrospective observational study	11	MEP	BEN	Clinical exacerbation, OCS dose, Eos, IgE, FeNO, FEV1, ACT
Walsh, L. J., 2022	Ireland	Article	2021.10–2022.2	Retrospective observational cohort study	20	RES	MEP 8, BEN 12	Clinical exacerbation, hospitalization, OCS dose, FEV1, ACQ
Li, Y., 2023	China	Article	2019.1–2022.5	Retrospective observational cohort study	10	OMA 9, BEN 1	DUP	Clinical exacerbation, OCS dose, ACT, FEV1, FeNO, Eos, IgE
Selvi, F. R., 2025	Italy	Article	2025.1–2025.6	Retrospective observational cohort study	15	BEN 7, OMA 4, MEP 4	DUP	Clinical exacerbation, OCS dose, FEV1, FeNO, Eos, IgE, ACT
Gates, J., 2025	United Kingdom	Article	2023.1–2024.2	Retrospective observational cohort study	98	IL‐5/R 82, OMA 12, DUP 4	TSLP	Clinical exacerbation, ACQ‐6, Eos, FeNO
Al Awn, L., 2025	Saudi Arabia	Article	2024.1—	Retrospective observational cohort study	33	OMA‐MEP 13; MEP‐DUP 10; OMA‐DUP 8		Clinical exacerbation, hospitalizations
Tran, T. N., 2025	23 countries enrolled in International Severe Asthma Registry (ISAR): USA 177, UK 124, Denmark 92, Kuwait 25, Australia 16, Canada 14, Italy 12, Spain 5, Saudi Arabia 4 etc.	Article	2015.12–2021.8	CLEAR: Multicenter retrospective observational cohort study	254	Not provided (mixed)		Clinical exacerbation, ER visit, hospitalizations, OCS dose
Sumi, T., 2025	Japan	Article	2022.12–2023.12	Retrospective observational cohort study	22	MEP/BEN 9, DUP 13	TSLP	OCS use, OCS dose, ACT, FEV1, IgE, FeNO, Eos

Abbreviations: ACT, asthma control test; ACQ, asthma control questionnaire; AQLQ, asthma quality of life questionnaire; BEN, benralizumab; DUP, dupilumab; Eos, eosinophil; ER, emergency room; FeNO, fractional exhaled nitric oxide; FEV1, forced expiratory volume in 1 s; IgE, immunoglobin E; MEP, mepolizumab; mOCS, maintenance oral corticosteroid; OMA, omalizumab; RES, reslizumab; TSLP, thymic stromal lymphopoietin.

The majority of included studies were deemed to be at low risk of bias, demonstrating high or moderate methodological quality assessment; however, two retrospective observational studies were assessed as having low quality in the domains of selection and comparability (Table 2). Leave‐one‐out sensitivity analysis indicated that no single study significantly influenced the MD for asthma‐related clinical outcomes (Figure S1). Visual inspection of the funnel plot, Egger's test, and fail‐safe N analysis indicated potential publication bias in the results of exacerbation rate and OCS dose; however, no evidence of small‐study effect was observed in all clinical outcomes (Data S2).

**TABLE 2:** Quality assessment of included studies in the systematic review and meta‐analysis.

First author, year	Country/region	Publication type	Study design	Assessment tool	Domain points	Quality
Akaba, T., 2023	Japan	Article	Retrospective observational cohort study	NOS	Selection score: 3/4 stars Comparability score: 0/2 stars Outcome score: 3/3 stars	Moderate
Cakmak, M. E., 2024	Türkiye	Article	Retrospective observational cohort study	NOS	Selection score: 4/4 stars Comparability score: 2/2 stars Outcome score: 3/3 stars	High
Cameli, P., 2024	Italy	Article	ANANKE (NCT04272463): Multicenter retrospective observational study	NOS	Selection score: 3/4 stars Comparability score: 0/2 stars Outcome score: 3/3 stars	Moderate
Caminati, M., 2023	Italy	Article	Multicenter retrospective observational study	NOS	Selection score: 3/4 stars Comparability score: 0/2 stars Outcome score: 2/3 stars	Moderate
Carpagnano, G. E., 2020	Italy	Article	Retrospective observational cohort study	NOS	Selection score: 3/4 stars Comparability score: 0/2 stars Outcome score: 3/3 stars	Moderate
Carstens, D., 2023	United States	Article	Retrospective observational cohort study	NOS	Selection score: 3/4 stars Comparability score: 1/2 stars Outcome score: 3/3 stars	High
Chapman, K. R., 2019	Argentina, Belgium, Canada, France, Germany, Netherlands, Spain, Sweden, United States	Article	OSMO (Omalizumab Switch to Mepolizumab study): Open‐label, single‐arm, multicenter trial (NCT02654145)	RoB 2, adapted for a single‐arm trial.	Bias arising from the randomization process: No information; Bias due to deviations from intended interventions: Some concerns; Bias due to missing outcome data: Low risk; Bias in measurement of the outcome: Some concerns; Bias in selection of the reported result: Low risk	Moderate (Some concerns)
Cheng, S. L., 2025	Taiwan	Article	REMIT: Retrospective observational cohort study	NOS	Selection score: 3/4 stars Comparability score: 2/2 stars Outcome score: 3/3 stars	High
Fernández, A. G. B., 2022	Spain	Article	Retrospective observational cohort study	NOS	Selection score: 2/4 stars Comparability score: 0/2 stars Outcome score: 3/3 stars	Moderate
Hashimoto, S.,2022	Netherlands	Article	Dutch severe asthma registry (RAPSODI): Multicenter retrospective observational cohort study	NOS	Selection score: 3/4 stars Comparability score: 1/2 star Outcome score: 3/3 stars	High
Higo, H., 2023	Japan	Article	Multicenter retrospective observational cohort study	NOS	Selection score: 3/4 stars Comparability score: 2/2 stars Outcome score: 1/3 stars	Moderate
Jackson, D., 2024	United Kingdom	Article	BPAP study (NCT05932849): Multicenter retrospective observational cohort study	NOS	Selection score: 3/3 stars Comparability score: 2/2 stars Outcome score: 3/3 stars	High
Jiménez‐Gómez, M., 2024	Spain	Article	Retrospective observational cohort study	NOS	Selection score: 2/4 stars Comparability score: 0/2 stars Outcome score: 3/3 stars	Moderate
Katran, Z. Y., 2023	Türkiye	Article	Retrospective observational cohort study	NOS	Selection score: 2/4 stars Comparability score: 2/2 stars Outcome score: 2/3 stars	Moderate
Lafarge, L., 2024	Belgium	Article	Monocenter retrospective observational cohort study	NOS	Selection score: 3/4 stars Comparability score: 0/2 stars Outcome score: 2/3 stars	Moderate
Mansur, A. H., 2023	United Kingdom	Article	UK Severe Asthma Registry (UKSAR): Retrospective observational cohort study	NOS	Selection score: 3/4 stars Comparability score: 0/2 stars Outcome score: 2/3 stars	Moderate
Naumova, V. V., 2023	Russia	Article	Retrospective observational cohort study	NOS	Selection score: 4/4 stars Comparability score: 1/2 stars Outcome score: 2/3 stars	High
Pelaia, C., 2021	Italy	Article	Multicenter retrospective observational cohort study	NOS	Selection score: 3/4 stars Comparability score: 2/2 stars Outcome score: 2/3 stars	High
Rosso, C., 2024	Italy	Article	Retrospective observational cohort study	NOS	Selection score: 2/4 stars Comparability score: 2/2 stars Outcome score: 2/3 stars	Moderate
Trevor, J., 2021	United States	Article	CHRONICLE (NCT03373045): Prospective observational cohort study	NOS	Selection score: 4/4 stars Comparability score: 2/2 stars Outcome score: 2/3 stars	High
Valery, S., 2024	France	Article	RAMSES cohort: Observational cohort study (Target trial emulation)	NOS	Selection score: 4/4 stars Comparability score: 2/2 stars Outcome score: 3/3 stars	High
Ais‐Daza, A., 2024	Spain	Conference	Ambispective observational cohort study	NOS	Selection score: 4/4 stars Comparability score: 0/2 stars Outcome score: 3/3 stars	High
Al‐Lehebi, R. O., 2022	Saudi Arabia	Conference	Retrospective observational cohort study	NOS	Selection score: 4/4 stars Comparability score: 0/2 stars Outcome score: 3/3 stars	High
Altous, M., 2021	Australia	Conference	Retrospective observational cohort study	NOS	Selection score: 4/4 stars Comparability score: 0/2 stars Outcome score: 2/3 stars	Moderate
Arismendi, E., 2024	Spain	Conference	Multicenter retrospective observational cohort study	NOS	Selection score: 4/4 stars Comparability score: 0/2 stars Outcome score: 3/3 stars	High
Bagnasco, D., 2019	Italy	Conference	Retrospective observational cohort study	NOS	Selection score: 4/4 stars Comparability score: 0/2 stars Outcome score: 3/3 stars	High
Corbridge, T., 2024	United States	Conference	Retrospective observational cohort study	NOS	Selection score: 4/4 stars Comparability score: 0/2 stars Outcome score: 3/3 stars	High
D'Amato, M., 2019	Italy	Conference	Retrospective observational cohort study	NOS	Selection score: 4/4 stars Comparability score: 1/2 stars Outcome score: 3/3 stars	High
Deb, A., 2023	United States	Conference	Retrospective observational cohort study	NOS	Selection score: 4/4 stars Comparability score: 0/2 stars Outcome score: 3/3 stars	High
Gates, J., 2023	United Kingdom	Conference	Retrospective observational cohort study	NOS	Selection score: 2/4 stars Comparability score: 0/2 stars Outcome score: 2/3 stars	Low
Jackson, D. J., 2024	United Kingdom	Conference	BPAP study: Retrospective observational cohort study	NOS	Selection score: 4/4 stars Comparability score: 0/2 stars Outcome score: 2/3 stars	Moderate
Naderisemiromi, M., 2023	United Kingdom	Conference	Retrospective observational cohort study	NOS	Selection score: 4/4 stars Comparability score: 0/2 stars Outcome score: 3/3 stars	High
Nopsopon, T., 2024	United States	Conference	Retrospective observational cohort study	NOS	Selection score: 3/4 stars Comparability score: 0/2 stars Outcome score: 2/3 stars	Moderate
Okawa, K., 2022	Japan	Conference	Retrospective observational cohort study	NOS	Selection score: 4/4 stars Comparability score: 1/2 stars Outcome score: 2/3 stars	High
Tavernier, G., 2021	United Kingdom	Conference	Prospective observational cohort study	NOS	Selection score: 3/4 stars Comparability score: 0/2 stars Outcome score: 2/3 stars	Moderate
Kallieri, M., 2024	Greece	Letter	RELIght: Multicenter prospective observational cohort study (post hoc analysis)	NOS	Selection score: 4/4 stars Comparability score: 1/2 stars Outcome score: 3/3 stars	High
O'reilly, E., 2022	Ireland	Letter	Retrospective observational cohort study	NOS	Selection score 3/4 stars Comparability score: 0/2 stars Outcome score: 3/3 stars	Moderate
Drick, N., 2020	Germany	Article	Multicenter retrospective observational cohort study	NOS	Selection score: 3/4 stars Comparability score: 0/2 stars Outcome score: 2/3 stars	Moderate
Kavanagh, J. E., 2021	United Kingdom	Letter	Retrospective observational case‐series study	NOS	Selection score: 3/4 stars Comparability score: 0/2 stars Outcome score: 3/3 stars	Moderate
Martinez‐Moragon, E., 2021	Spain	Article	ORBE study: Multicenter retrospective observational cohort study	NOS	Selection score: 3/4 stars Comparability score: 0/2 stars Outcome Ycore: 2/3 stars	Moderate
Mummler, C., 2021	Germany	Article	Retrospective observational cohort study	NOS	Selection score: 4/4 stars Comparability score: 1/2 stars Outcome score: 3/3 stars	High
Numata, T., 2020	Japan	Article	Retrospective observational study	NOS	Selection score: 3/4 stars Comparability score: 0/2 stars Outcome score: 2/3 stars	Moderate
Walsh, L. J., 2022	Ireland	Article	Retrospective observational cohort study	NOS	Selection score: 4/4 stars Comparability score: 0/2 stars Outcome score: 3/3 stars	High
Li, Y., 2023	China	Article	Retrospective observational cohort study	NOS	Selection score: 1/4 stars Comparability score: 0/2 stars Outcome score: 3/3 stars	Low
Selvi, F. R., 2025	Italy	Article	Retrospective observational cohort study	NOS	Selection score: 3/4 stars Comparability score: 0/2 stars Outcome score: 3/3 stars	Moderate
Gates, J., 2025	United Kingdom	Article	Retrospective observational cohort study	NOS	Selection score: 3/4 stars Comparability score: 0/2 stars Outcome score: 3/3 stars	Moderate
Al Awn, L., 2025	Saudi Arabia	Article	Retrospective observational cohort study	NOS	Selection score: 4/4 stars Comparability score: 0/2 stars Outcome score: 2/3 stars	Moderate
Tran, T. N., 2025	23 countries enrolled in International Severe Asthma Registry (ISAR): USA 177, UK 124, Denmark 92, Kuwait 25, Australia 16, Canada 14, Italy 12, Spain 5, Saudi Arabia 4 etc.	Article	CLEAR: Multicenter retrospective observational cohort study	NOS	Selection score: 4/4 stars Comparability score: 2/2 stars Outcome score: 2/3 stars	High
Sumi, T., 2025	Japan	Article	Retrospective observational cohort study	NOS	Selection score: 2/4 stars Comparability score: 0/2 stars Outcome score: 3/3 stars	Moderate

*Note:* Green indicates high quality, blue indicates mediated quality, and red indicates low quality.

### 3.2 The Switching Patterns

**FIGURE 2:**
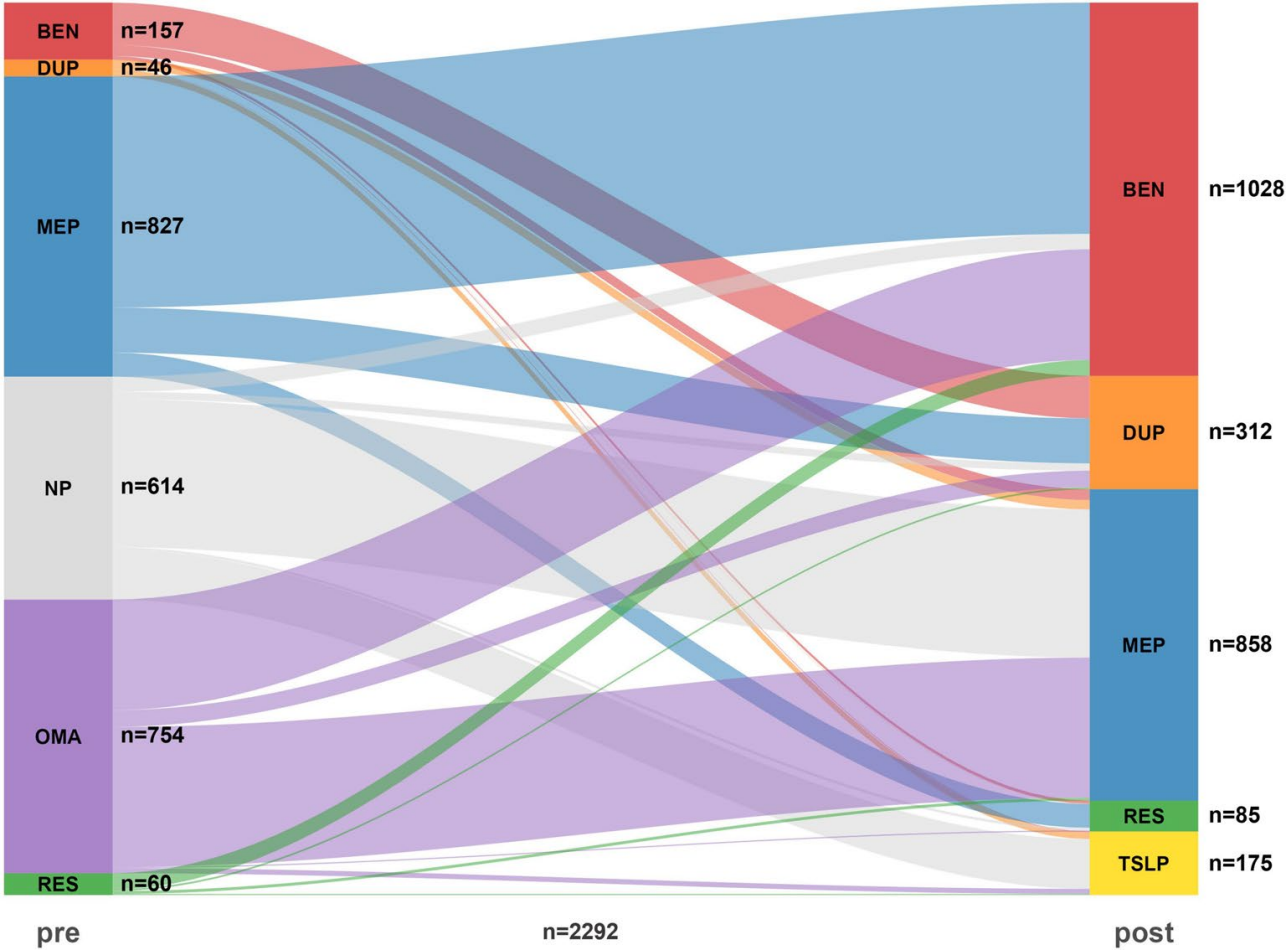
Sankey plot illustrating biologic switches in meta‐analysis. The source nodes on the left side represent initial categories and numbers of biologic use prior to switch; each node's height is proportional to total quantity of flow originating from it. The target nodes on the right side represent final categories and numbers of biologic use after switch. The flows connecting the source and target nodes represent different reported biologic switch choices, with mepolizumab‐benralizumab (*n* = 637), omalizumab‐mepolizumab (*n* = 386), and omalizumab‐benralizumab (*n* = 305) being the most common ones.

A Sankey diagram (Figure 2) was created to show the patterns of biologics switch. The study reports on 2292 documented switched severe asthma patients, with 614 cases (27%) where the specific biologic type is not reported. Prior to the switch, mepolizumab (*n* = 827) and omalizumab (*n* = 754) were the most commonly used biologics, while post‐switch benralizumab (*n* = 1028) and mepolizumab (*n* = 858) were the most commonly used biologics. Mepolizumab‐benralizumab (*n* = 637), omalizumab‐mepolizumab (*n* = 386), omalizumab‐benralizumab (*n* = 305) were the most commonly encountered switch patterns.

**FIGURE 3:**
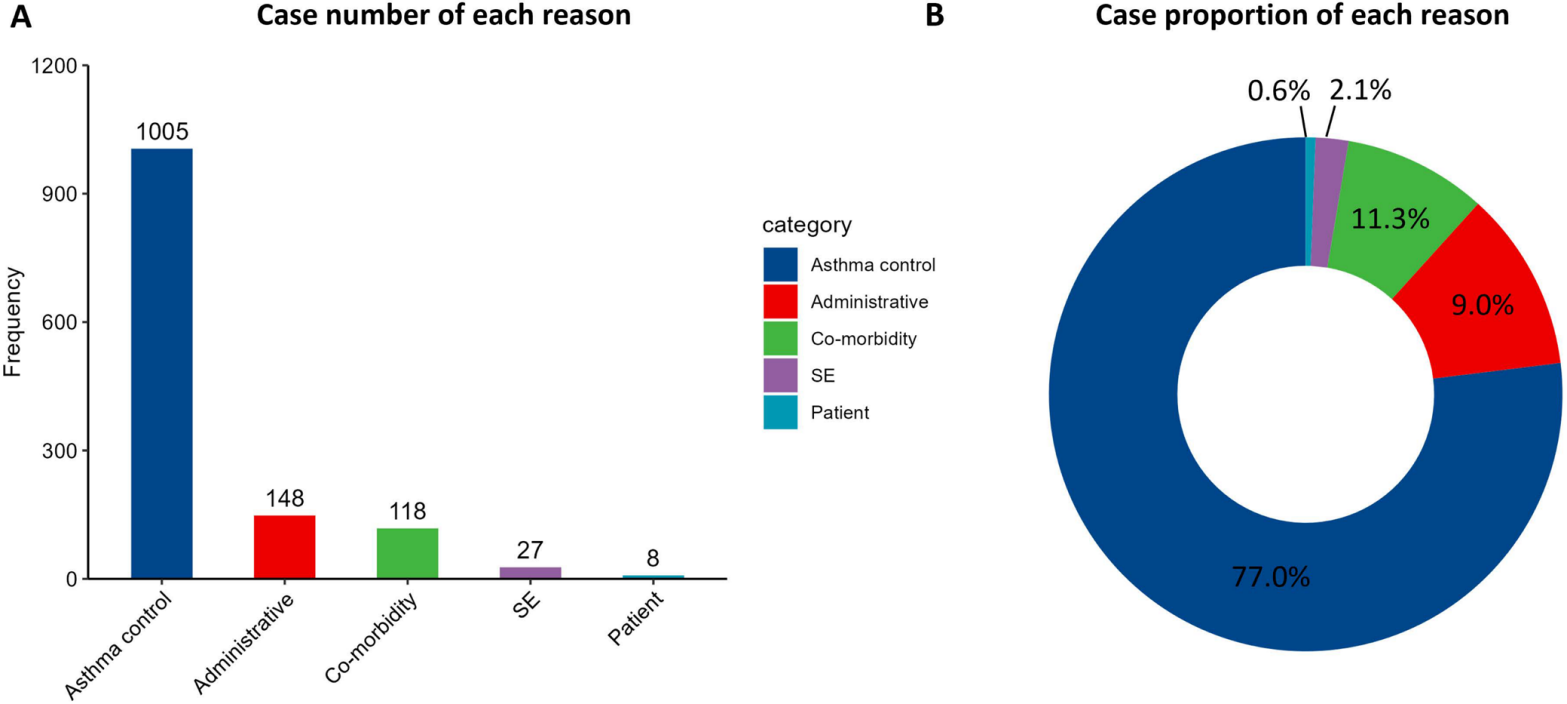
Reasons for biologics switch. (A) Barplots showing the frequency of different reasons for biologic switch. (B) Pie chart showing the proportional distribution of different reasons. Categories are defined as follows: Suboptimal response of asthma control; Suboptimal response of asthma co‐morbidities control; SE, side effect; Patient reasons, including self‐administration or request, convenience; Administrative matters, including clinical trial in a manner that reflects clinical practice.

Suboptimal response of asthma control (*n* = 1005, 77.0%) was the most common reason for biologics switch (Figure 3). Other reasons included: (a) administrative reasons (*n* = 148, 12.0%) including organization switch, doctor's choice, clinical trial in a manner reflecting clinical practice; (b) suboptimal response of co‐morbidities (*n* = 118, 9.0%) including allergic rhinitis, chronic rhinosinusitis with nasal polyps, eosinophilic otitis media, etc.; (c) biologics‐related side effect (*n* = 27, 2.2%); (d) patient preferences (*n* = 6, 0.5%) including the choice of self‐administration, patient request to change, convenience.

### 3.3 The Impact of the Switch on Clinical Remission

**FIGURE 4:**
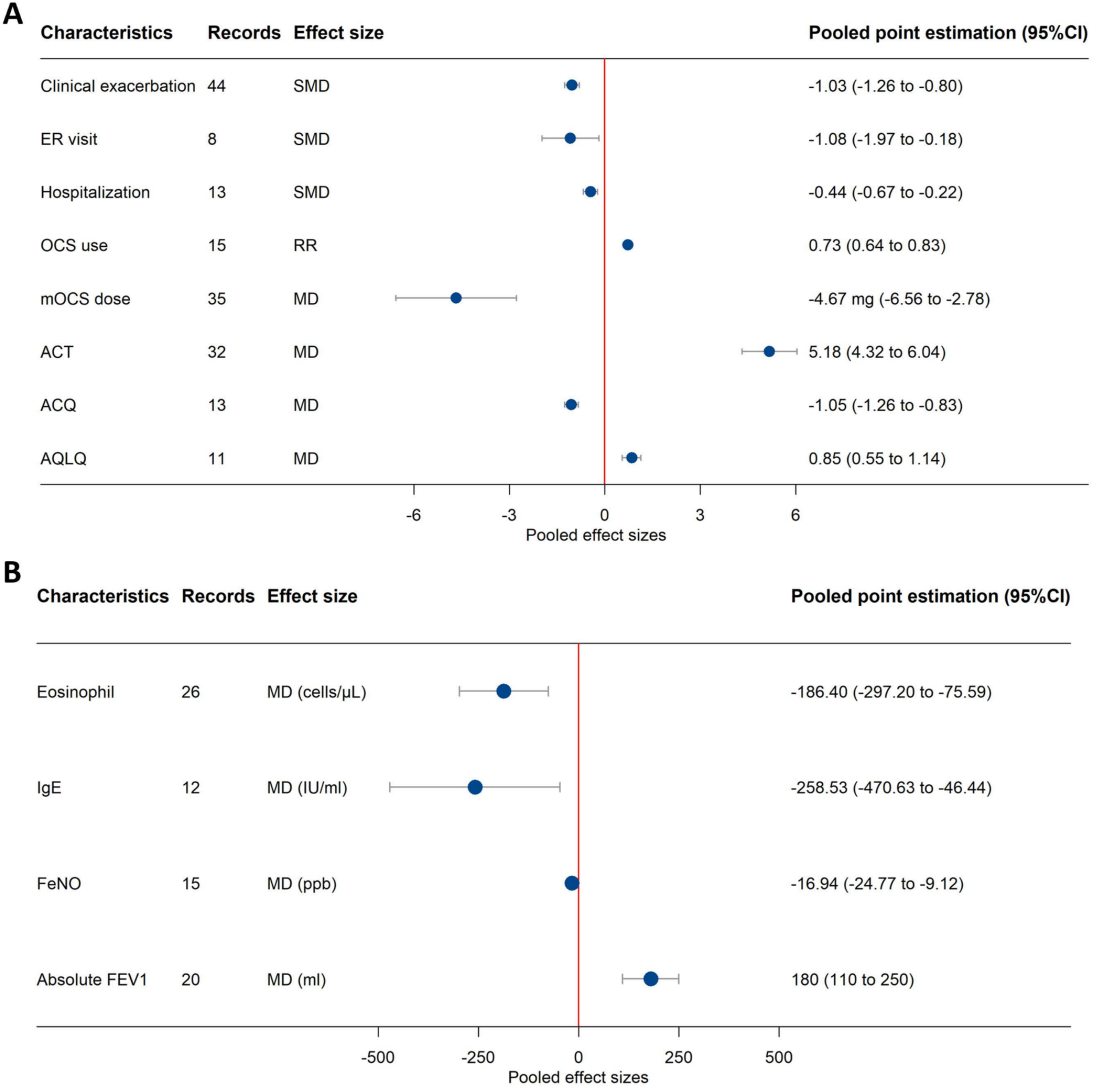
Estimation of pooled effect sizes (MD, SMD, RR) between pre‐ and post‐biologic switches in meta‐analysis. (A) Clinical and symptomatic outcomes, including clinical exacerbation, ER visit, hospitalization, OCS use, and clinical questionnaires scores (ACT, ACQ, AQLQ). (B) Laboratory indicators, including eosinophil count, IgE levels, FeNO, and absolute FEV1. Circles and lines represent pooled point estimates and 95% confidence intervals (CIs) of effect sizes. MD, mean difference; RR, relative risk; SMD, standardized mean difference.

Figure 4 summarizes the key findings of the meta‐analysis. For each clinical outcome assessed, the pooled point estimate along with its 95% confidence interval (CI) is displayed.

### 3.4 Component–Asthma Exacerbation Rate

For exacerbation rate 44 reports showed a significant improvement after biologics switch (SMD = −1.03, 95% CI: −1.26 to −0.80, *I* ^2^ = 89%). In the subgroup analysis of different post‐switch biologics types, the switch effect varied by the types of biologic administered post‐switch, with the SMD ranging from −1.38 (95% CI −1.94 to −0.82, *I* ^2^ = 79%) for dupilumab (*n* = 9), −1.04 (95% CI −1.46 to −0.62, *I* ^2^ = 92%) for benralizumab (*n* = 17), −1.01 (95% CI −1.53 to −0.49, *I* ^2^ = 89%) for mepolizumab (*n* = 9), and −0.70 (95% CI −1.12 to −0.28, *I* ^2^ = 60%) for TSLP (*n* = 3) (Figure S2A). The post hoc sensitivity analysis was performed by excluding data from conference abstracts, which showed a significant improvement in exacerbation rate (SMD = −0.98, 95% CI −1.18 to −0.78, *I* ^2^ = 78%, *n* = 34) after biologics switch (Figure S2B). Emergency department visits were reduced significantly after biologics switch (SMD = −1.08, 95% CI: −1.97 to −0.18, *I* ^2^ = 87%). The calculation included the outcomes reported by 8 studies. In the subgroup analysis of different post‐switch biologics types, the SMD of the switch impact varied from −2.61 (95% CI: −6.77 to 1.54) to −0.29 (95% CI: −0.45 to −0.13). The post hoc sensitivity analysis based on the publication type consistently supported a significant reduction in emergency department visits with a SMD of −0.55 (95% CI −0.81 to −0.28, *I* ^2^ = 48%, *n* = 6) (Figure S3A,B).

Hospitalizations' rate reported by 13 studies showed a significant improvement after biologics switch (SMD = −0.44, 95% CI: −0.67 to −0.22, *I* ^2^ = 57%). In the subgroup analysis of different post‐switch biologics types, the SMD of the switch impact varied from −0.62 (95% CI: −1.12 to −0.11, *I* ^2^ = 21%) to −0.34 (95% CI: −0.80 to 0.12, *I* ^2^ = 79%). Post hoc sensitivity analysis also showed a significant reduction in hospitalization rate with a SMD of −0.40 (95% CI –0.65 to −0.15, *I* ^2^ = 57%, *n* = 11) (Figure S3C,D).

### 3.5 Component‐Maintenance OCS

Fifteen studies reported the percentage of severe asthma patients using OCS. The pooled prevalence of OCS use before switching was 60.35% (95% CI: 42.39–77.06, *I* ^2^ = 95.8%), significantly decreasing to 33.14% after the switch (95% CI: 16.98–51.49, *I* ^2^ = 96%) according to a relative risk (RR) of 0.73 (95% CI: 0.64–0.83, *I* ^2^ = 59%). The post hoc sensitivity analysis stratified by publication type confirmed a significant reduction in OCS use, with an RR of 0.74 (95% CI: 0.64–0.85; *I* ^2^ = 61%) (Figure S4A,B). Maintenance dose of OCS also decreased significantly after the switch, with an MD of −4.67 mg (95% CI: −6.56 to −2.78, *I* ^2^ = 94%). Subgroup analysis by types of post‐switched biologics showed MDs ranging from −5.55 mg (95% CI: −9.44 to −1.66) for benralizumab to −0.10 mg (95% CI: −5.96 to 3.96) for tezepelumab. A post hoc sensitivity analysis by the type of publication yielded consistent results for maintenance OCS dose reduction (Figure S4C,D).

### 3.6 Component‐Asthma Control

Pooled data from 32 studies reporting ACT scores indicate a significant improvement after the switch, with a MD of 5.18 points (95% CI: 4.32–6.04, *I* ^2^ = 80%). Subgroup analysis by post‐switched biologic types revealed modest variability, with MDs ranging from 3.77 (95% CI: 2.60–4.93) to 5.83 (95% CI: 4.30–7.36). Pooled data from 13 studies reporting ACQ scores, showed a significant decline in the ACQ score after the switch, with an MD of −1.05 points (95% CI: −1.26 to −0.83, *I* ^2^ = 45%). Subgroup analysis by different biologics demonstrated variability, with MDs ranging from −0.77 points (95% CI: −1.03 to −0.50) to −1.65 points (95% CI: −2.62 to −0.68). Pooled data from 11 studies reporting AQLQ scores, pooled data indicated an improvement in the QoL after the switch with an MD of 0.85 points (95% CI: 0.55 to 1.14, *I* ^2^ = 70%). Subgroup analysis by different biologics showed variability, with MDs ranging from 0.20 (95% CI: −0.19 to 0.59) to 1.26 (95% CI: 0.30–2.21). Post hoc sensitivity analysis, excluding publication types of conference abstracts, yielded consistent results for ACT, ACQ, and AQLQ score changes (Figure S5).

### 3.7 Component‐Lung Function

Prebronchodilator FEV1 reported in 39 studies was used as an outcome measure for the impact of the switch on pulmonary function in our analysis. Pooled data from 20 studies reporting absolute FEV1 showed an improvement with an MD of 0.18 L (95% CI: 0.11–0.25, *I* ^2^ = 0%), with subgroup analysis indicating variability ranging from 0.04 L (95% CI: −0.29 to 0.37) for TSLP to 0.26 L (95% CI: 0.10–0.41) for dupilumab. Pooled data from 19 studies reporting FEV1% showed an increase of MD = 8.73% (95% CI 5.15–12.30, *I* ^2^ = 82%), with subgroup analysis indicating variability ranging from 6.19% (95% CI: 1.02–11.36) for mepolizumab to 17.00% (95% CI: 11.37–22.62) for dupilumab (Figure S6).

### 3.8 The Impact of the Switch on T2‐Biomarkers

Blood eosinophil counts were reported in 26 studies, total serum IgE in 12, and FeNO in 15 studies. Pooled data indicated a reduction in blood eosinophil counts after the switch with an MD of −186.40 cells/μL (95% CI: −297.20 to −75.59, *I* ^2^ = 96%). Subgroup analysis by biologic types revealed notable variability, with dupilumab and TSLP being associated with an increase in blood eosinophil counts, with MDs of 99.16 cells/μL (95% CI: −66.55 to 264.87) and 62.79 cells/μL (95% CI: 26.16 to 99.41), respectively; in contrast, with the other biologics that induced a reduction in blood eosinophil counts with an MD of −279.50 cells/μL (95% CI: −391.81 to −167.20). Total serum IgE levels decreased with an MD of −258.53 IU/mL (95% CI: −470.63 to −46.44, *I* ^2^ = 92%), and subgroup analysis by biologic types showed variability, with MDs ranging from −1363 IU/mL (95% CI: −1673.52 to −1052.48) to 24.53 IU/mL (95% CI: −39.34 to 88.41). FeNO levels demonstrated a reduction with an MD of −16.94 ppb (95% CI: −24.77 to −9.12, *I* ^2^ = 82%), with subgroup analysis indicating variability ranging from −30.34 ppb (95% CI: −39.45 to −21.24) for dupilumab to −6.10 ppb (95% CI: −12.81 to 0.61) for mepolizumab. The results of the post hoc sensitivity analysis of publication type were consistent for T2 biomarkers changes (Figure S7).

## 4 Discussion

Advances in understanding of the epithelial barrier dysfunction [24] and T2‐high severe asthma endotype [25] have positioned biologics as a pivotal therapeutic option [26]. Current guidelines recommend initiating biologics in severe asthma after evaluating asthma control, OCS use, T2 biomarkers, comorbidities, and patient preferences [11, 27, 28, 29]. A treatment duration of 4–6 months is typically needed to assess the effectiveness of a biologic agent [11, 27]. If there is no response, a switch to other biologics targeting the T2 pathways is recommended, after careful evaluation of the reason for non‐response [28]. Adding a second biologic is currently not recommended due to high costs and insufficient evidence [27]. Despite these advances in severe asthma treatment, real‐world data on the efficacy, safety, and factors influencing biologics switching in patients with severe asthma remain relatively scarce, which highlighted the need for more high‐quality real‐world evidence [14]. Collectively, our systematic review and meta‐analysis provides a comprehensive and quantitative synthesis of the clinical efficacy of biologic switching, supported by high‐quality evidence‐based data, which could address critical gaps in current guidelines.

To our knowledge, this study is the first to comprehensively synthesize the evidence from observational studies and clinical trials, delineating the patterns and rationales for biologic switching in patients with severe asthma, while evaluating its efficacy across all components of clinical remission criteria, including exacerbations, asthma control, quality of life, pulmonary function, OCS use and T2‐biomarkers. We report here on major novel insights on the switch impact. First, all clinical remission outcomes improved following a biologic switch. Clinical remission, characterized by lack of exacerbations, improved asthma control, discontinuation of OCS use, and improved lung function, has been proposed as an important treatment goal for patients with severe asthma [30]. The SR primary findings suggested that all components of clinical remission were achieved following a biologic switch, with particularly robust evidence for reductions in exacerbations (44 studies) and improvement in asthma control (32 studies). Additionally, these improvements were clinically meaningful, as the pooled MD for ACT (5.18 points, MCID = 3 points), ACQ (−1.05 points, MCID = –0.5 points), and AQLQ (0.85 points, MCID = 0.5 points) all exceed their respective minimal clinically important differences (MCIDs) [31, 32]. Subgroup analyses by biologic type revealed that, while different biologics achieved comparable clinical efficacy, subtle variations in specific clinical outcomes were observed, possibly related to their distinct mechanisms of action. For example, dupilumab exhibited the greatest efficacy in improving lung function (FEV1) and in decreasing FeNO but had no impact on blood eosinophils. Subgroup variations were also contextualized against these MCID thresholds, demonstrating favorable outcomes in most subgroups across different biologics.

The SR results indicated anti‐IgE to anti‐IL‐5/5R and one anti‐IL‐5/5R biologic to another as prevalent switching strategies. Anti‐IL‐5/5R biologics were the most frequently reported and the predominant post‐switch choice, while anti‐IgE biologics were exclusively documented as a pre‐switch option. This switching pattern may be partly attributed to the historical timeline of biologic approvals. Anti‐IgE (omalizumab) was the first approved biologic for severe asthma in patients aged ≥ 6 years in 2003 in Europe and the United States, which had achieved nearly 2 million patient‐years of exposure [33]. Its efficacy and safety, supported by numerous real‐world studies, post hoc analyses of clinical trials, and systematic reviews, enabled omalizumab's implementation as preferred initial add‐on biologic therapy in clinical practice [34]. Subsequently, the development and approval of anti‐IL5/5R, anti‐IL4/IL‐13, and anti‐TSLP biologics over the past decade had expanded treatment options for severe asthma [35]. Previous studies have reported that the type of biologic might impact achieving clinical remission of asthma, with anti‐IL5/5R and anti‐IL4/IL‐13 demonstrating superior therapeutic outcomes compared with anti‐IgE [36]. Beyond clinical remission, co‐morbidities/treatable traits is another vital determinant in switching choice. For example, non‐responders to omalizumab with uncontrolled CRSwNP can be switched to anti‐IL‐4/IL‐13 or to anti‐IL5/5R therapies; non‐responders to anti‐IL5/5R therapies can be switched to anti IL‐4/IL‐13 if they have uncontrolled AD, allergic rhinitis, or CRSwNP; alternatively, they may switch to anti‐IgE therapies for concurrent uncontrolled chronic spontaneous urticaria or severe food allergy. Other factors influencing switching include biologics‐related side effects, medical costs, local payer eligibility criteria, availability, administration route, and patient preferences, as observed in our analysis [37].

**FIGURE 5:**
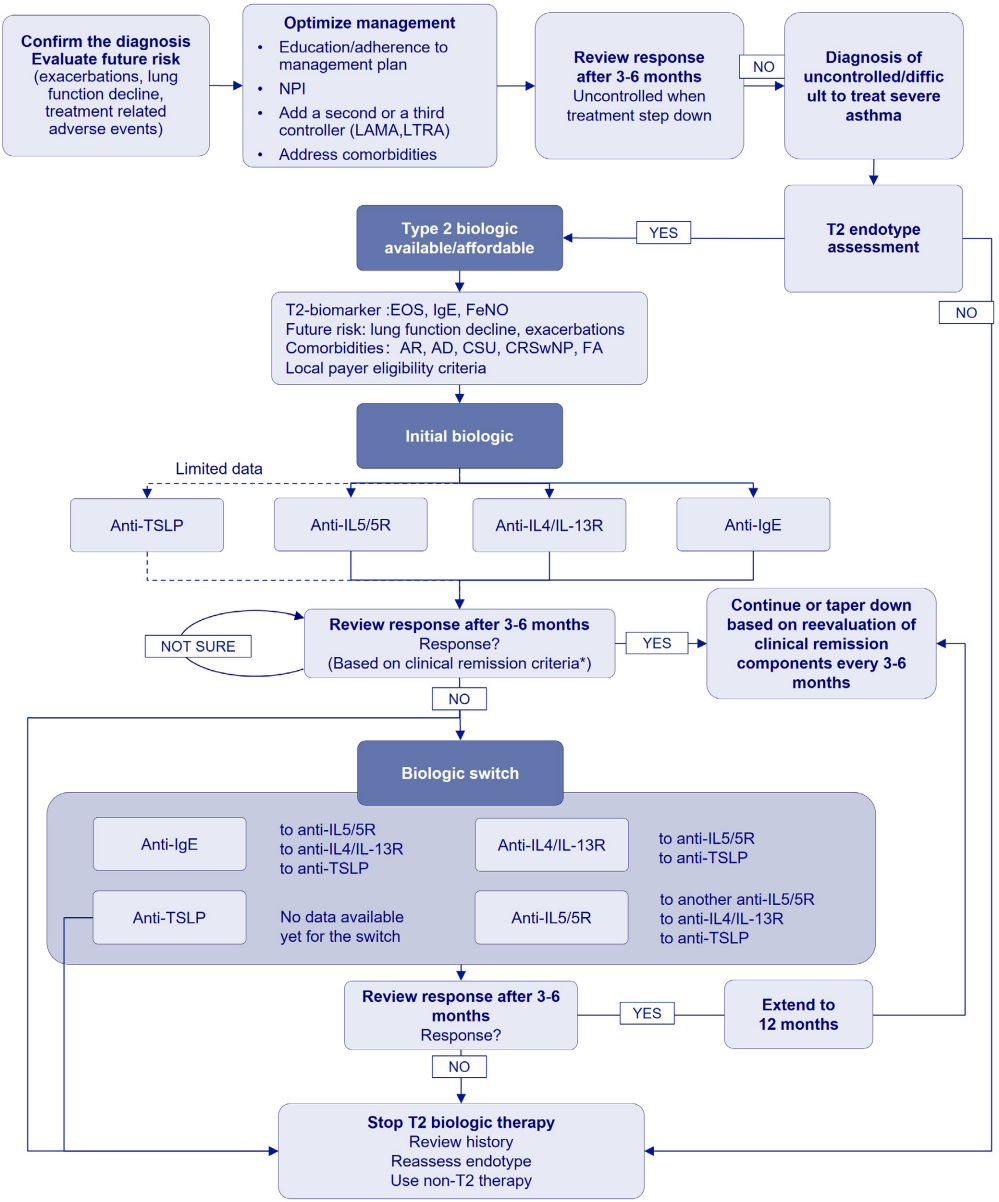
Algorithm to guide the biologics switch in patients with severe asthma. After confirmation of the diagnosis of severe uncontrolled/difficult to treat asthma, the T2 high status should be assessed by using the available biomarkers (e.g., blood eosinophils ≥ 300/μL on two separate occasions, FeNO > 30 ppb, sputum eosinophils > 2.5%). The choice of the initial biologic is based on the association with specific co‐morbidities/treatable traits (e.g., allergic sensitization, food allergy, or chronic urticaria for omalizumab; very high levels of blood eosinophils for anti‐IL‐5/5R interventions; atopic dermatitis, CRSwNP or lung function decline for anti‐IL‐4/IL‐13 interventions) and local payer eligibility criteria. Treatment response is assessed based on four‐component clinical remission criteria: (1) very mild or no asthma symptoms (e.g., ACT ≥ 20 or ≥ 23, ACQ < 1.5 or ≤ 0.75 for very mild or no symptoms, respectively), (2) no asthma exacerbations, (3) no use of systemic corticosteroids, and (4) stable or normal lung function (e.g., FEV1 decline of ≤ 5% or FEV1 ≥ 80% predicted) (Cited from *Lommatzsch M*, *Virchow JC. Lancet Respir Med. 2025*). For lack of response, switching biologics is indicated based on unachieved components of clinical remission and on the associated co‐morbidities/treatable traits. For example, non‐responders to omalizumab with persistent low lung function can be switched to anti‐IL‐4/IL‐13 or to anti‐IL5/5R targeted interventions if they have persistent high levels of blood eosinophils; non‐responders to dupilumab can be switched to anti‐IL5 targeted interventions if they have persistent high levels of blood eosinophils or if they develop dupilumab related ocular adverse; non‐responders to anti‐IL5/5R targeted interventions can be switched to anti IL‐4/IL‐13 if they have persistent low lung function or if they have uncontrolled AD or CRSwNP or to another biological targeting the IL‐5/5R pathway but with a different mode of action (anti IL‐5 to anti IL‐5R) or allowing increased dosage (SC to IV for example with reslizumab) for persistent high blood eosinophils. Non‐T2 therapy includes LAMA, low dose azithromycin, epithelial barrier restoration, bronchial thermoplasty targeting airway smooth muscle, bitter taste/olfactory receptors agonists, anti‐ IL‐33/ST2, anti‐IL‐17 or inflammasome targeted interventions, JAK inhibitors, HDAC inhibitors or other interventions aiming to counteract steroid resistance. AD, atopic dermatitis; CRSwNP, chronic rhinosinusitis with nasal polyps; EOS, eosinophils; FA, Food allergy; FeNO, fractional exhaled nitric oxide; FEV_1_, forced expiratory volume in 1 s; HDAC, histone deacetylase; ICS‐LABA, inhaled corticosteroid‐ long‐acting β_2_‐agonist; IgE, immunoglobulin E; IL, interleukin; JAK, Janus kinase; LAMA, long‐acting muscarinic antagonist; NPI, non‐pharmacological intervention; OCS, oral corticosteroids; ST2, suppression of tumorigenicity 2/interleukin‐1 receptor‐like 1; TSLP, thymic stromal lymphopoietin.

Overall, the prevalent biologic switching patterns identified in this study provide valuable guidance for future clinical practice. Accordingly, we synthetize our observations into an algorithm to guide the biologics switch in patients with severe asthma (Figure 5). However, a personalized approach [38, 39] to biologic selection should consider multiple factors, including treatment response, side effects, cost, and patient preferences to optimize outcomes.

This SR has several limitations that warrant consideration. First, the meta‐analysis may be affected by the heterogeneity arising from the publication type. The inclusion of conference abstracts in the pooled analyses remains controversial due to concerns over methodological rigor and quality. However, given the rapid evolution of biologic therapies for asthma, a substantial volume of contemporary data is disseminated exclusively as conference abstracts. Excluding this publication format would probably omit valuable evidence. Accordingly, we incorporated all publication types—including full articles, letters, and conference abstracts—while rigorously assessing conference abstract quality and conducting post hoc sensitivity analyses to explore the impact of abstract data inclusion. Second, publication bias and small‐study effects may have influenced our results (e.g., asthma exacerbation rate, proportion of OCS use, mOCS dose), potentially leading to overestimation or underestimation of pooled effect sizes. Notably, the number of records varied markedly across biologic classes, with anti‐IL‐5/5R and anti‐IgE agents comprising the majority of included studies. This underscores a cautious interpretation of these findings and highlights the value of larger, confirmatory trials. Third, the limited and variable FEV_1_ criteria used to define treatment failure before biologic switching preclude a standardized classification of treatment failure and may introduce heterogeneity in patient selection and baseline severity, potentially influencing the generalizability of observed improvements in lung function. Fourth, clinical outcomes were assessed at multiple time points ranging from several months to 1 year. Consistent with guidelines recommendations [11], we selected the assessment timepoint closest to the 1‐year mark for primary analyses. Fifth, data are driven primarily by observational studies, with only one RCT included. There is still an unmet need for prospective RCTs to assess clinical remission between different biologics [40].

## 5 Conclusion

Biologics switching represents a promising strategy for patients with severe asthma, supported by high‐quality evidence of clinical efficacy. Switching should be personalized, considering factors such as treatment response, side effects, cost, and patient preferences to optimize outcomes.

## Author Contributions

**Yang Zheng:** investigation, methodology, project administration, writing – original draft preparation, writing – review and editing. **Ya‐chun Li:** investigation, methodology, project administration, writing – original draft preparation. **Sheng‐jie Li:** investigation, methodology, project administration. **Meng Xu:** investigation, methodology, project administration. **Jia‐qian Hu:** investigation, methodology, project administration. **Wen‐qu Tian:** investigation, methodology, project administration. **Shi‐wei Chen:** investigation, methodology, project administration. **Xue‐hui Li:** investigation, methodology, project administration. **Ying He:** investigation, methodology, project administration. **Gan Lu:** investigation, methodology, project administration. **Mübeccel Akdis:** supervision, writing – review and editing. **Ioana Agache:** supervision, writing – review and editing. **Cezmi Akdis:** supervision, writing – review and editing. **Ya‐dong Gao:** conceptualization, investigation, methodology, project administration, supervision, writing – original draft preparation, writing – review and editing.

## Funding

This work was supported by the National Natural Science Foundation of China (grant number 72204214), Zhejiang Provincial Natural Science Foundation of China (grant number LTGY24H260001, LQN25H030006, and LQN25H020010), Start‐up Research fund by The First Affiliated Hospital of Zhejiang University School of Medicine (BQD 2306), Zhejiang Clinovation Pride (grant number CXTD202501015), and Horizontal research project from the Suzhou Collaborative Medicine Foundation on Diagnosis and Treatment of Moderate‐to‐Severe Asthma (Phase II) (grant number Z001).

## Ethics Statement

The authors have nothing to report.

## Consent

The authors have nothing to report.

## Conflicts of Interest

C. A. Akdis has received research grants from the Swiss National Science Foundation, European Union (EU CURE, EU Syn‐Air‐G), Novartis Research Institutes, (Basel, Switzerland), Stanford University (Redwood City, Calif), Seed Health (Boston, USA) and SciBase (Stockholm, Sweden); is the Co‐Chair for EAACI Guidelines on Environmental Science in Allergic diseases and Asthma; Chair of the EAACI Epithelial Cell Biology Working Group is on the Advisory Boards of Sanofi/Regeneron (Bern, Switzerland, New York, USA), Stanford University Sean Parker Asthma Allergy Center (CA, USA), Novartis (Basel, Switzerland), Glaxo Smith Kline (Zurich, Switzerland), Bristol‐Myers Squibb (New York, USA), Seed Health (Boston, USA) and SciBase (Stockholm, Sweden); and is the Editor‐in‐Chief of Allergy. I. Agache reports being the Deputy Editor of Allergy. M. Akdis has received research grants from Swiss National science Foundation, Bern; research grant from the Stanford University; Leading House for the Latin American Region, Seed Money Grant. She is on the Scientific Advisory Board member of Stanford University‐Sean Parker Asthma Allergy Center, CA; Advisory Board member of LEO Foundation Skin Immunology Research Center, Kopenhagen; and Scientific Co‐Chair of World Allergy Congress (WAC) Istanbul, 2022, Scientific Programme Committee Chair, EAACI. Other authors declare no conflicts of interest.

## Supporting information


**Data S1:** Search strategies.
**Figure S1:** Leave‐one‐out sensitivity analysis.
**Figure S2:** Publication bias and small‐study effect.
**Figure S3:** Forest plots of clinical outcomes.
**Figure S2:** Asthma exacerbation rate (A. All publication types, B. excluding publication type of conference abstract).
**Figure S3:** ER visit and hospitalization.
**Figure S4:** OCS.
**Figure S5:** ACT, ACQ, AQLQ.
**Figure S6:** FEV1.
**Figure S7:** T2‐biomarkers.

## Data Availability

All data generated or analyzed during this study are included in this published article and its Supporting Information—S1 files.
