# Supplementary material for: Patterns and Clinical Efficacy of Biologics Switching in Patients With Severe Asthma: A Systematic Review and Meta‐Analysis
Source: Allergy. 2026 Apr 4;81(5):1548–70. doi: 10.1111/all.70333 (PMC13139823; doi:10.1111/all.70333)
Supplement: Supplementary file 1 — Data S1: Search strategies. Figure S1: Leave‐one‐out sensitivity analysis. Figure S2: Publication bias and small‐study effect. Figure S3: Forest plots of clinical outcomes. Figure S2: Asthma exacerbation rate (A. All publication types, B. excluding publication type of conference abstract). Figure S3: ER visit and hospitalization. Figure S4: OCS. Figure S5: ACT, ACQ, AQLQ. Figure S6: FEV1. Figure S7: T2‐biomarkers. [file ALL-81-1548-s001.docx]

Supplementary material to **Patterns and clinical efficacy of biologics switching in patients with severe asthma: a systematic review and meta-analysis**

**Supplemental S1. Search strategies**

**Pubmed/MEDLINE**

#1 mepolizumab[Title/Abstract] OR benralizumab[Title/Abstract] OR omalizumab[Title/Abstract] OR dupilumab[Title/Abstract] OR IL-4[Title/Abstract] OR IL-5[Title/Abstract] OR IL-13[Title/Abstract] OR IgE[Title/Abstract] OR Tezepelumab[Title/Abstract] OR TSLP[Title/Abstract])

#2 severe asthma[Title/Abstract] OR severe eosinophilic asthma[Title/Abstract] OR eosinophi* [Title/Abstract]

#3 (biologic*[Title/Abstract] OR antibody[Title/Abstract] ) AND switch*[Title/Abstract]

#1 AND #2 AND #3

**EMBASE**

#1 'mepolizumab'/exp OR mepolizumab OR 'benralizumab'/exp OR benralizumab OR 'omalizumab'/exp OR omalizumab OR 'dupilumab'/exp OR dupilumab OR 'il 4'/exp OR 'il 4' OR 'il 5'/exp OR 'il 5' OR 'il 13'/exp OR 'il 13' OR 'immunoglobulin e'/exp OR 'immunoglobulin e' OR 'Tezepelumab' OR 'TSLP'

#2 'severe asthma' OR 'severe eosinophilic asthma' OR 'eosinophi*'

#3 ('biologic*' OR 'antibody') AND 'switch*'

#1 AND #2 AND #3

**Supplemental Figure 1. Leave-one-out sensitivity analysis**


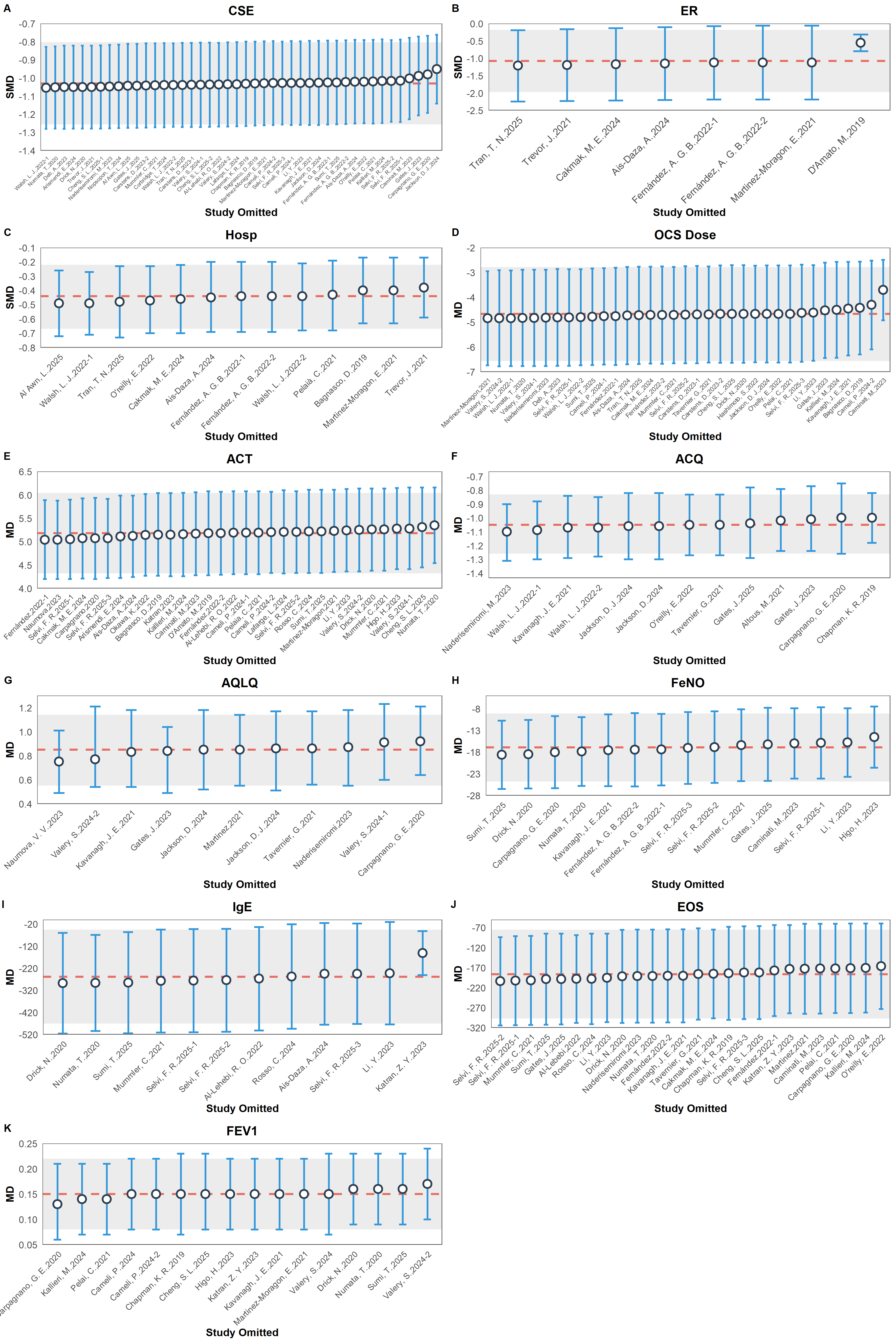


Leave-one-out sensitivity analyses for 11 outcomes in biologic switch. Points with blue error bars show effect estimates when each study is omitted. Red dashed line and gray area show 95% CI of overall effect.

**Supplemental S2. Publication bias and small-study effect**

Asthma exacerbation: Funnel plot and trim-and-fill


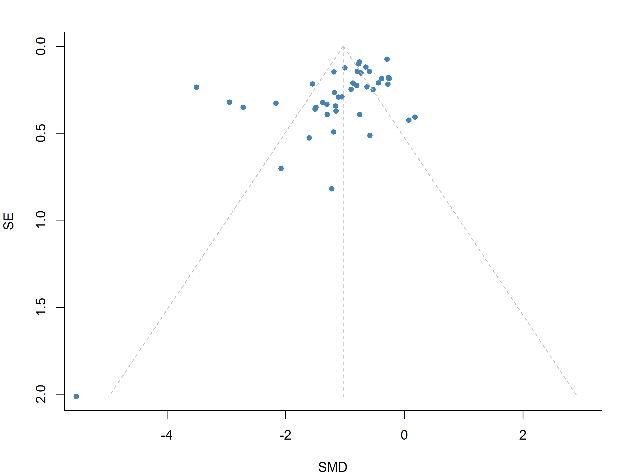

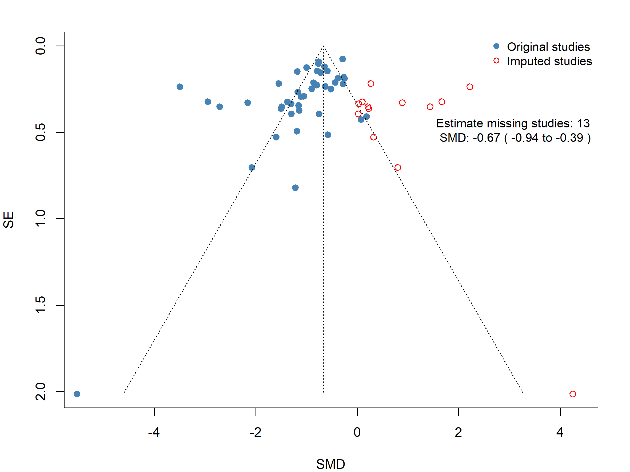


**This meta-analysis revealed evidence of small-study effects (Egger's test: t = -3.082, p = 0.004), suggesting potential publication bias, with smaller studies tending to report larger effect sizes which may have led to overestimation of the true treatment effect. However, the robustness of our findings is supported by trim-and-fill method and fail-saft number analysis. The trim-and-fill adjustment imputed 13 potentially missing studies and maintained a statistically significant pooled effect (SMD = -0.67, 95% CI: -0.94 to -0.39), indicating that the biologics-switching’s effectiveness persists after accounting for funnel plot asymmetry. The Rosenthal fail-safe N analysis yielded a value of 11,815, exceeding conventional recommended thresholds of 5k+10 and suggesting minimal susceptibility to publication bias.**

ER visit:


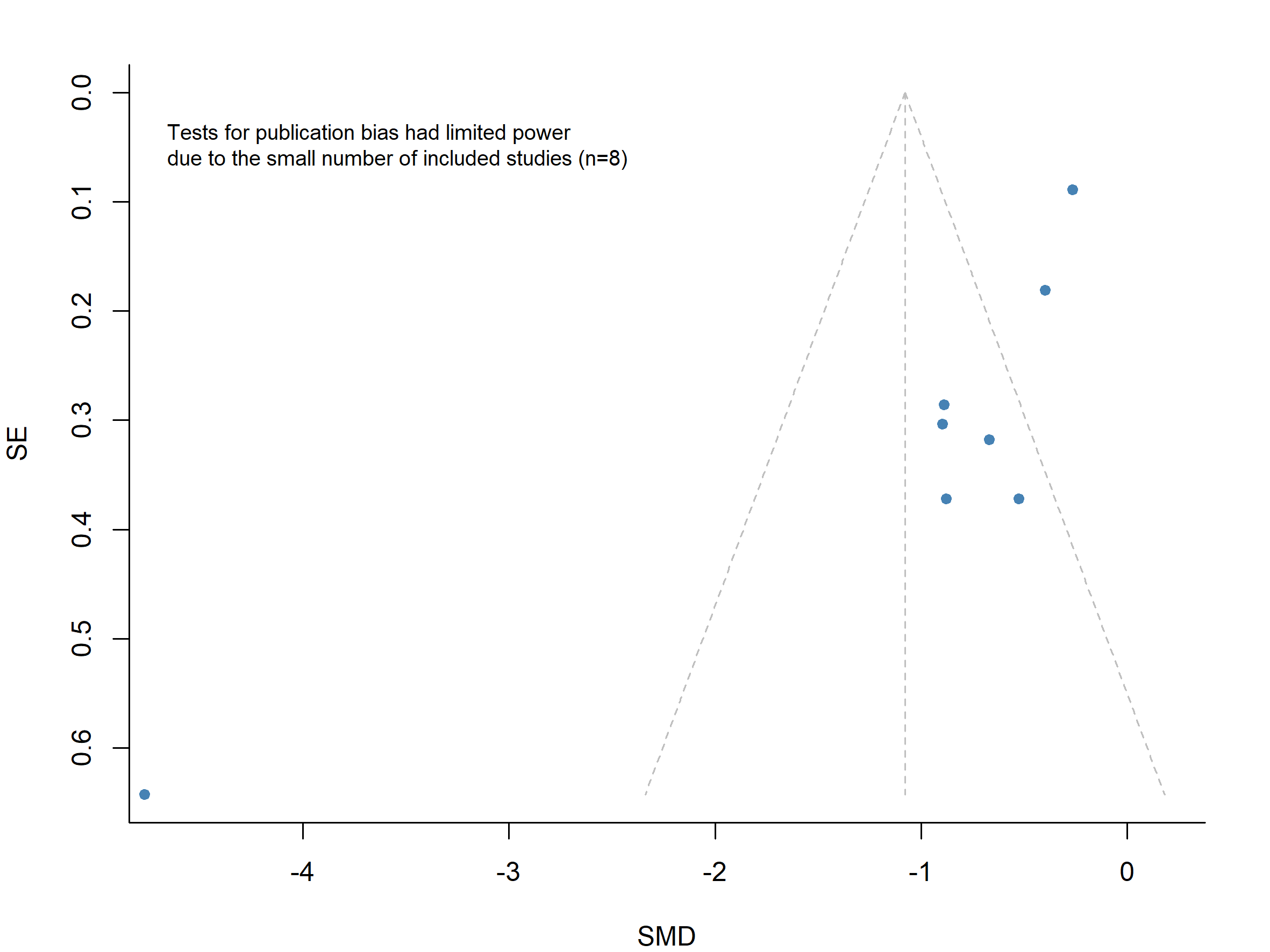


Tests for publication bias had limited power due to the small number of included studies <10. **The Rosenthal fail-safe N analysis yielded a value of 214, exceeding conventional recommended thresholds of 5k+10 and suggesting minimal susceptibility to publication bias.**

Hospitalization


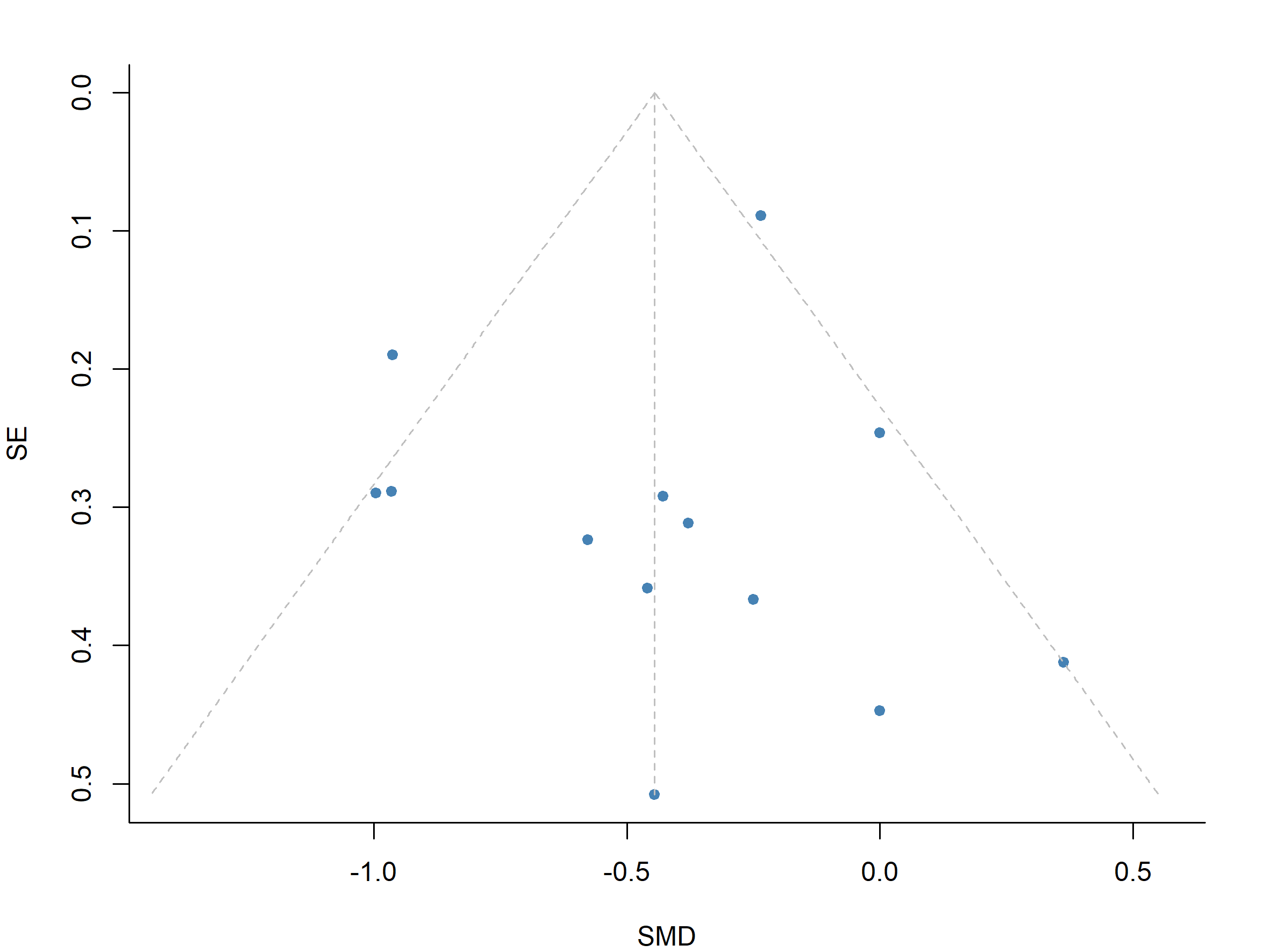


**Both visual inspection of the funnel plot and Egger's test result (t = -0.611, p = 0.554) indicated no evidence of small-study effects or publication bias.**

**Proportion of OCS use**


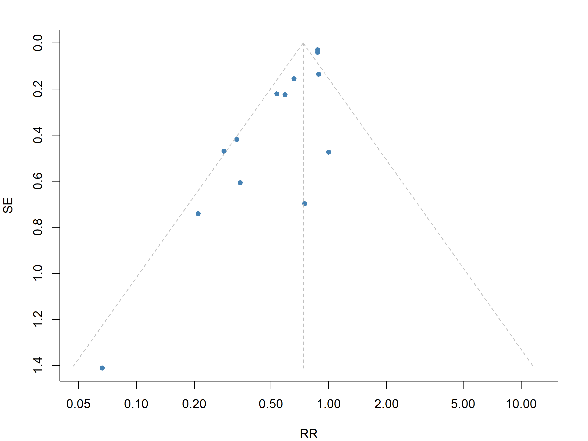

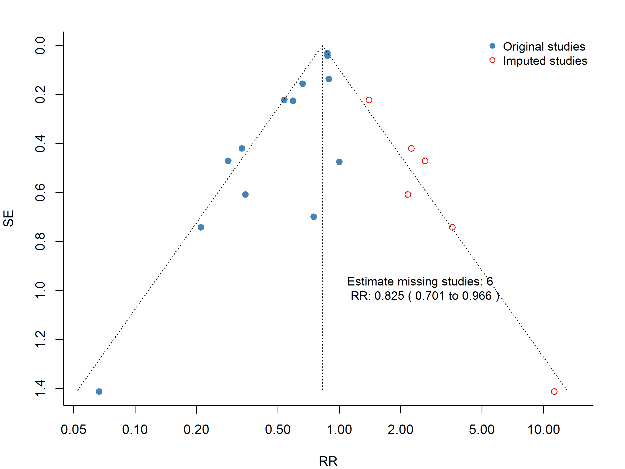


**This meta-analysis revealed evidence of small-study effects (Egger's test: t = -4.92, p = 0.001), suggesting potential publication bias, with smaller studies tending to report larger effect sizes which may have led to overestimation of the true treatment effect. The trim-and-fill adjustment imputed 6 potentially missing studies and remained statistically significant and consistent in direction with the original estimate (RR = 0.825, 95% CI: 0.701 to 0.966). Although the Rosenthal fail-safe N value of 271 exceeds conventional thresholds of 5k+10, suggesting robustness against unpublished null studies, the attenuation of the point estimate indicates vulnerability to other forms of small-study effects that have over-estimated the point estimate.**

**maintenance OCS dose**


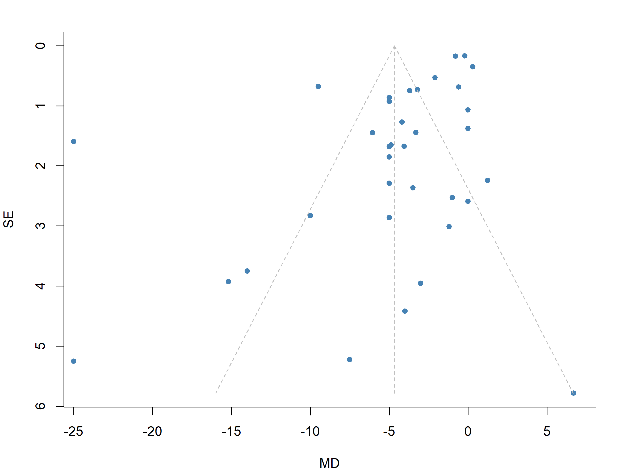

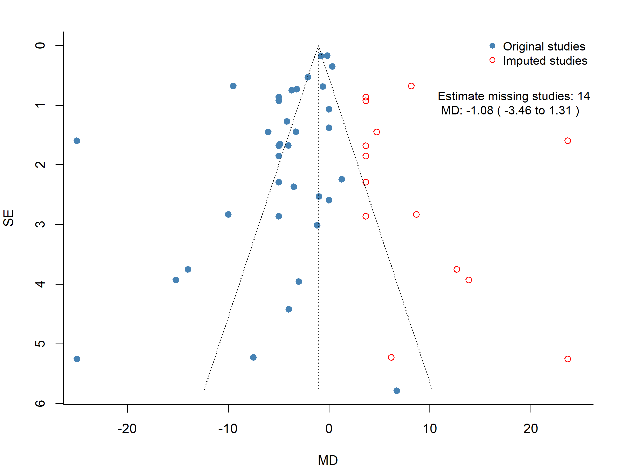


**This meta-analysis revealed evidence of small-study effects (Egger's test: t = -3.69, p = 0.001), suggesting potential publication bias, with smaller studies tending to report larger effect sizes which may have led to overestimation of the true treatment effect. The trim-and-fill adjustment imputed 14 potentially missing studies and attenuated the treatment effect to non-significance (WMD = -1.08, 95% CI: -3.46 to 1.31). Although the Rosenthal fail-safe N value of 3985 exceeds conventional thresholds of 5k+10, suggesting robustness against unpublished null studies, the attenuation of the point estimate indicates vulnerability to other forms of small-study effects that have over-estimated the point estimate.**

ACT


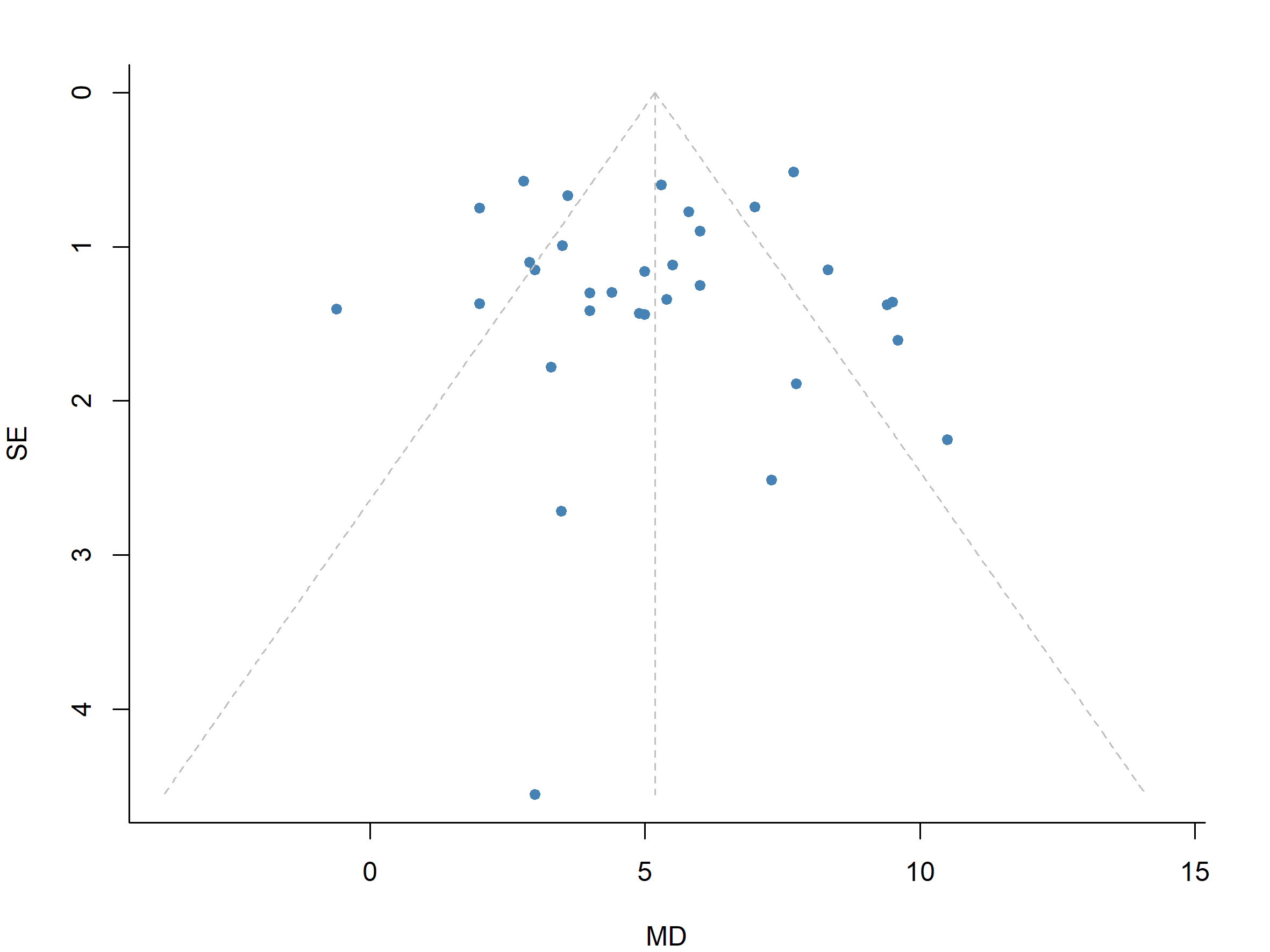


**Both visual inspection of the funnel plot and Egger's test result (t = 0.215 , p = 0.832) indicated no evidence of small-study effects or publication bias.**

ACQ


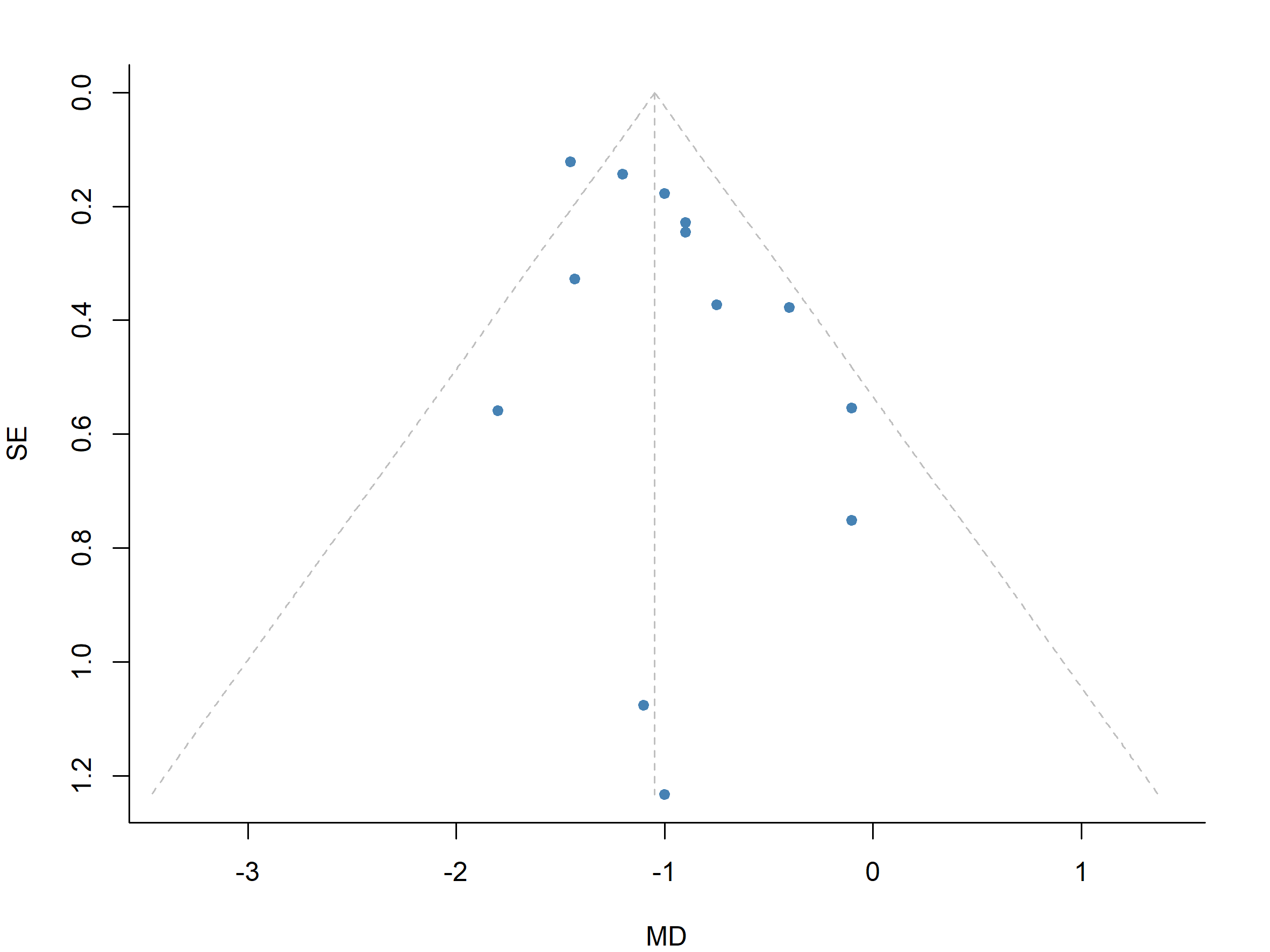


**Both visual inspection of the funnel plot and Egger's test result (t = 1.921 , p = 0.081) indicated no evidence of small-study effects or publication bias.**

AQLQ


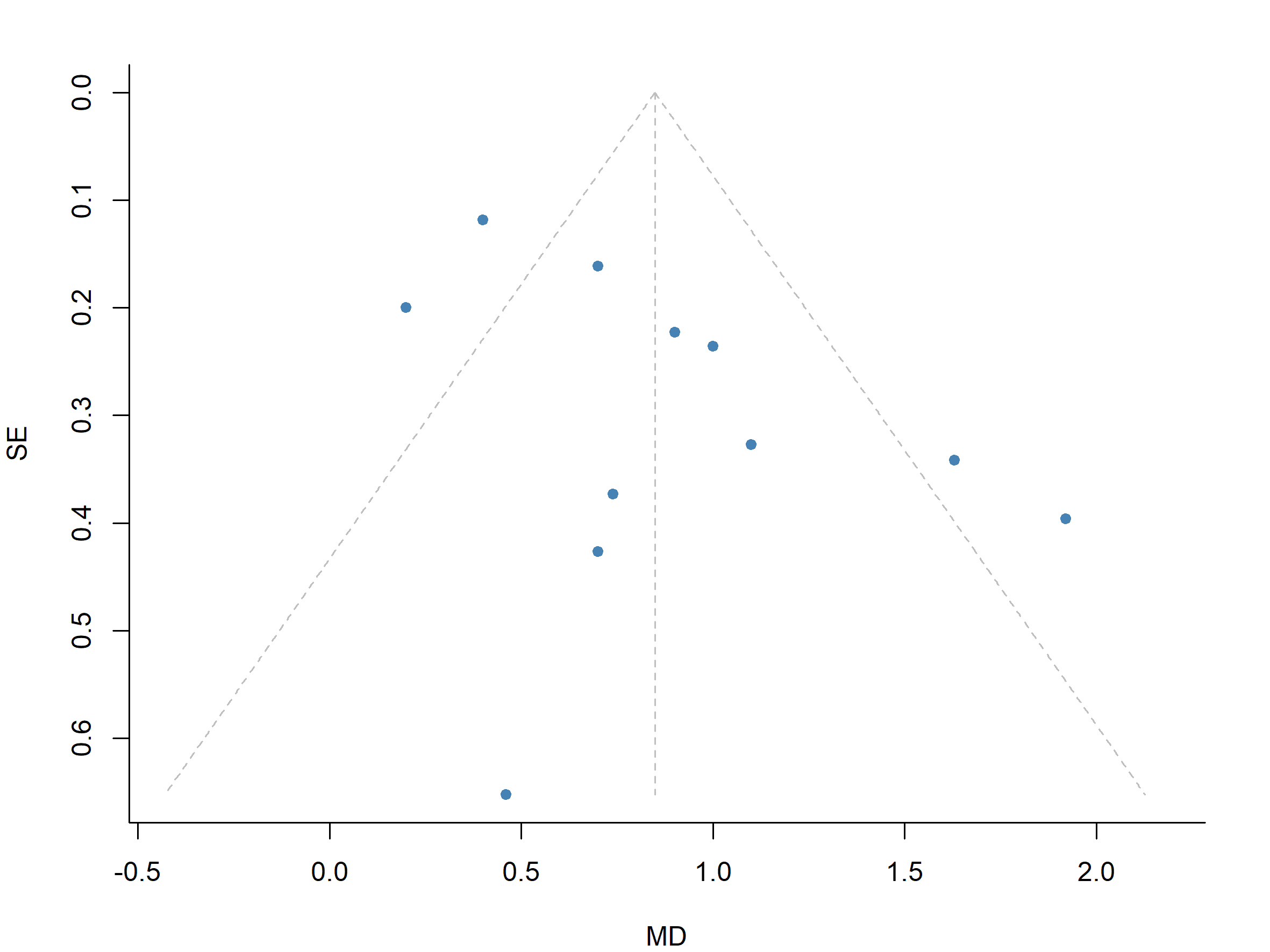


**Both visual inspection of the funnel plot and Egger's test result (t = 2.204 , p = 0.055) indicated no evidence of small-study effects or publication bias.**

FENO


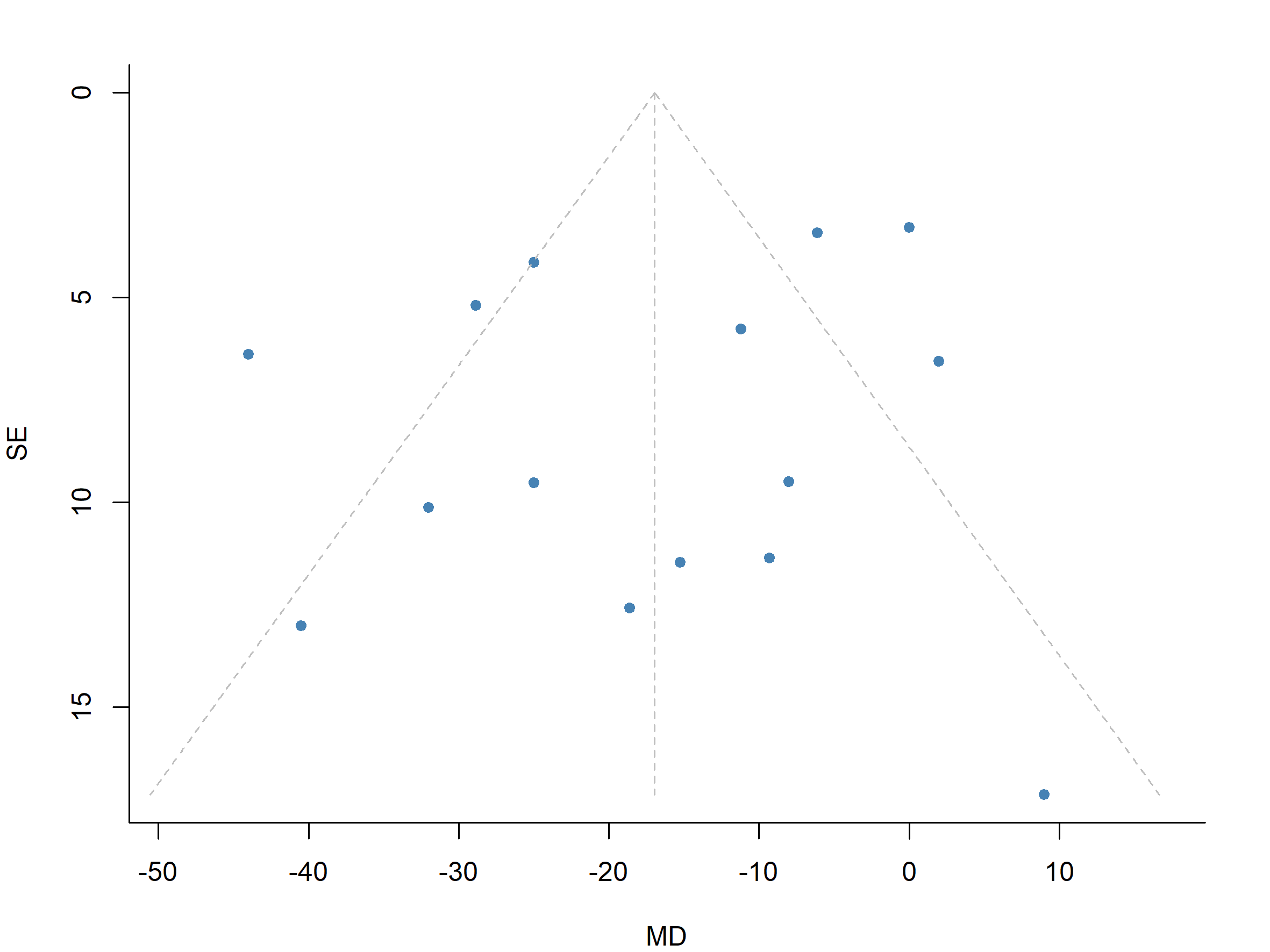


**Both visual inspection of the funnel plot and Egger's test result (t = -1.145 , p = 0.273) indicated no evidence of small-study effects or publication bias.**

IgE


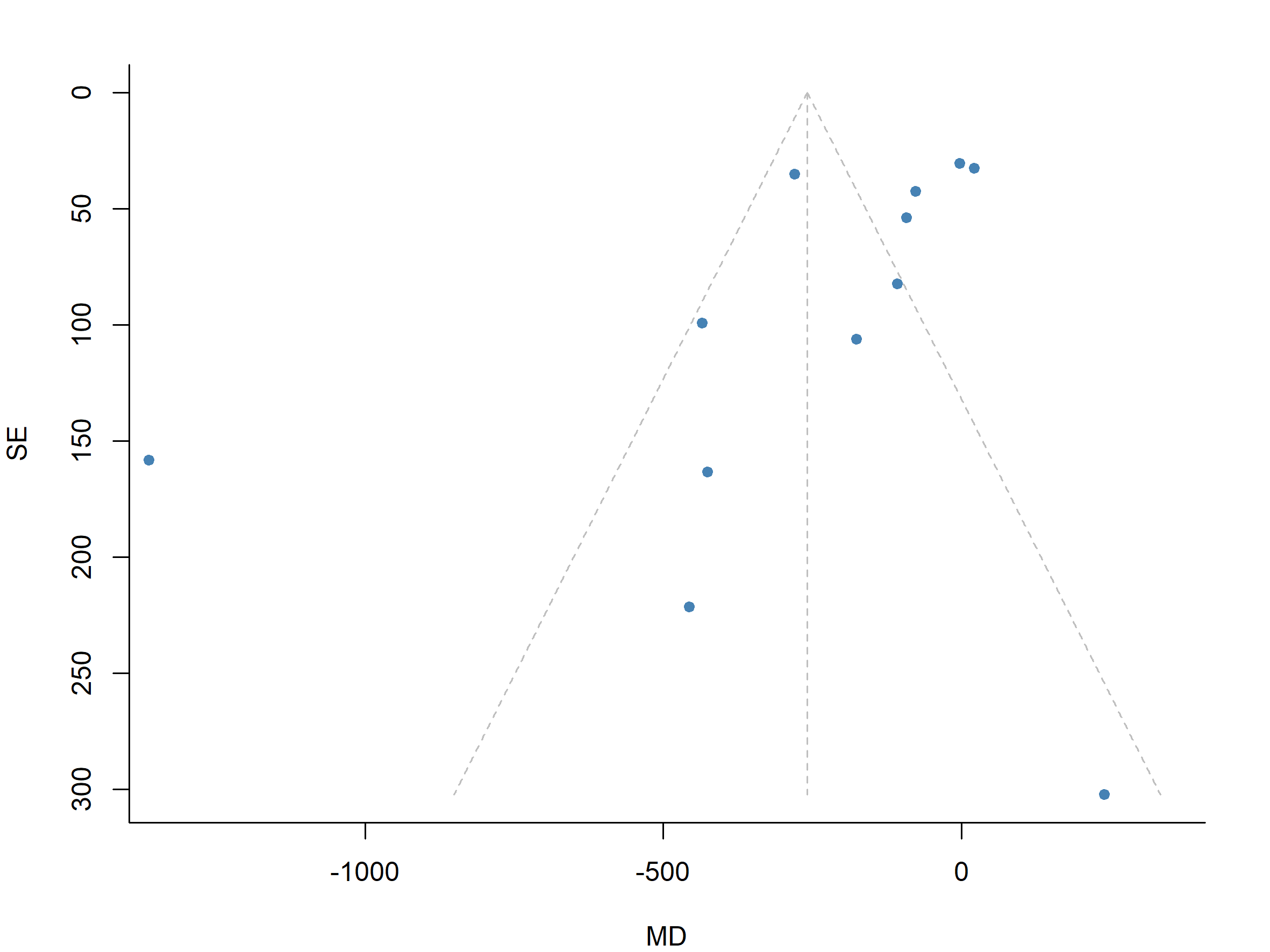


**Both visual inspection of the funnel plot and Egger's test result (t = -1.852 , p = 0.094) indicated no evidence of small-study effects or publication bias.**

Eosinophil


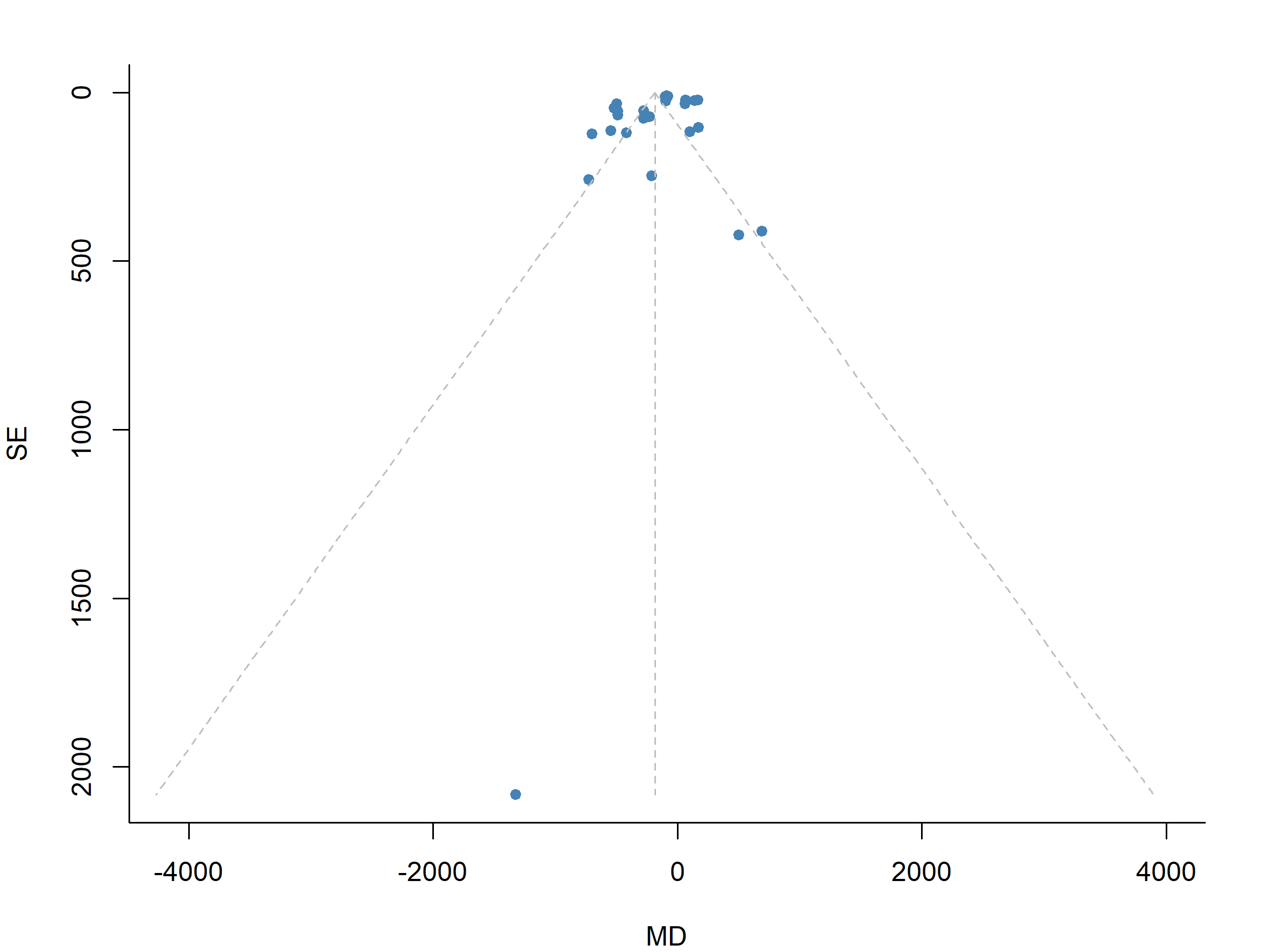


**Both visual inspection of the funnel plot and Egger's test result (t = -1.301 , p = 0.206) indicated no evidence of small-study effects or publication bias.**

FEV1


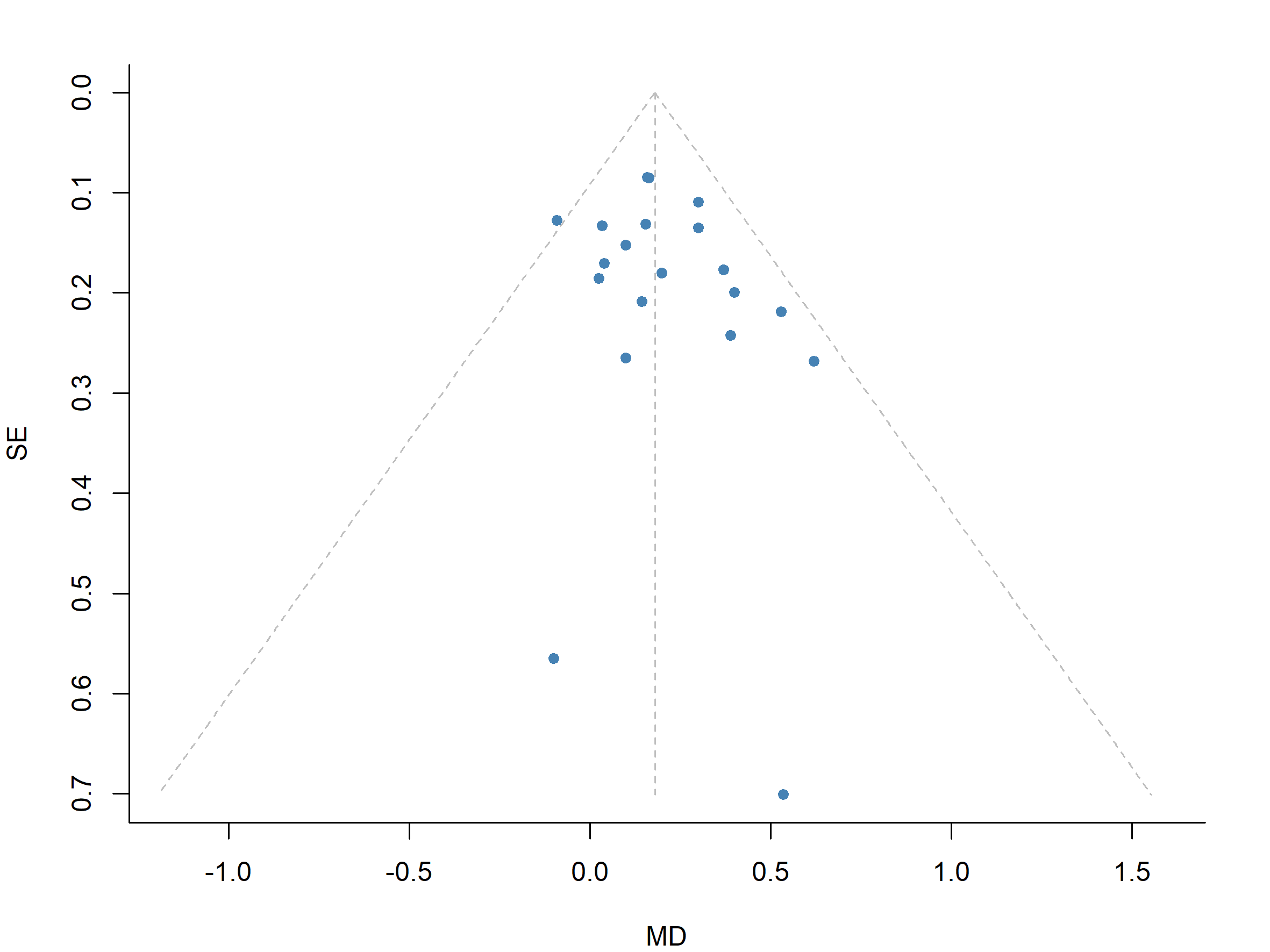


**Both visual inspection of the funnel plot and Egger's test result (t = -1.301 , p = 0.206) indicated no evidence of small-study effects or publication bias.**

**Supplemental S3. Forest plots of clinical outcomes**


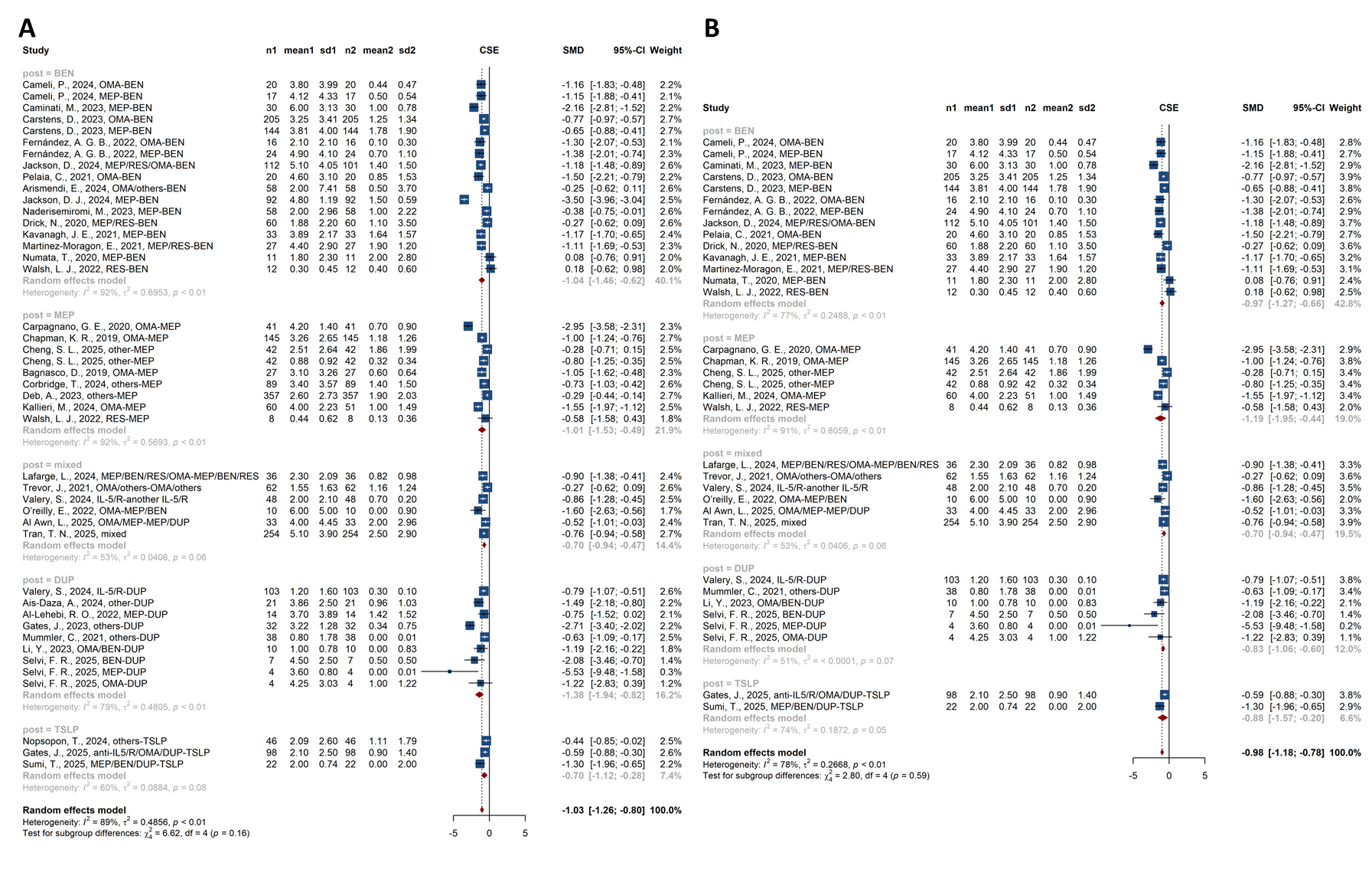
**Supplemental Figure 2. Asthma exacerbation rate (A. All publication types, B. excluding publication type of conference abstract)**

**Supplemental Figure 3. ER visit and hospitalization**


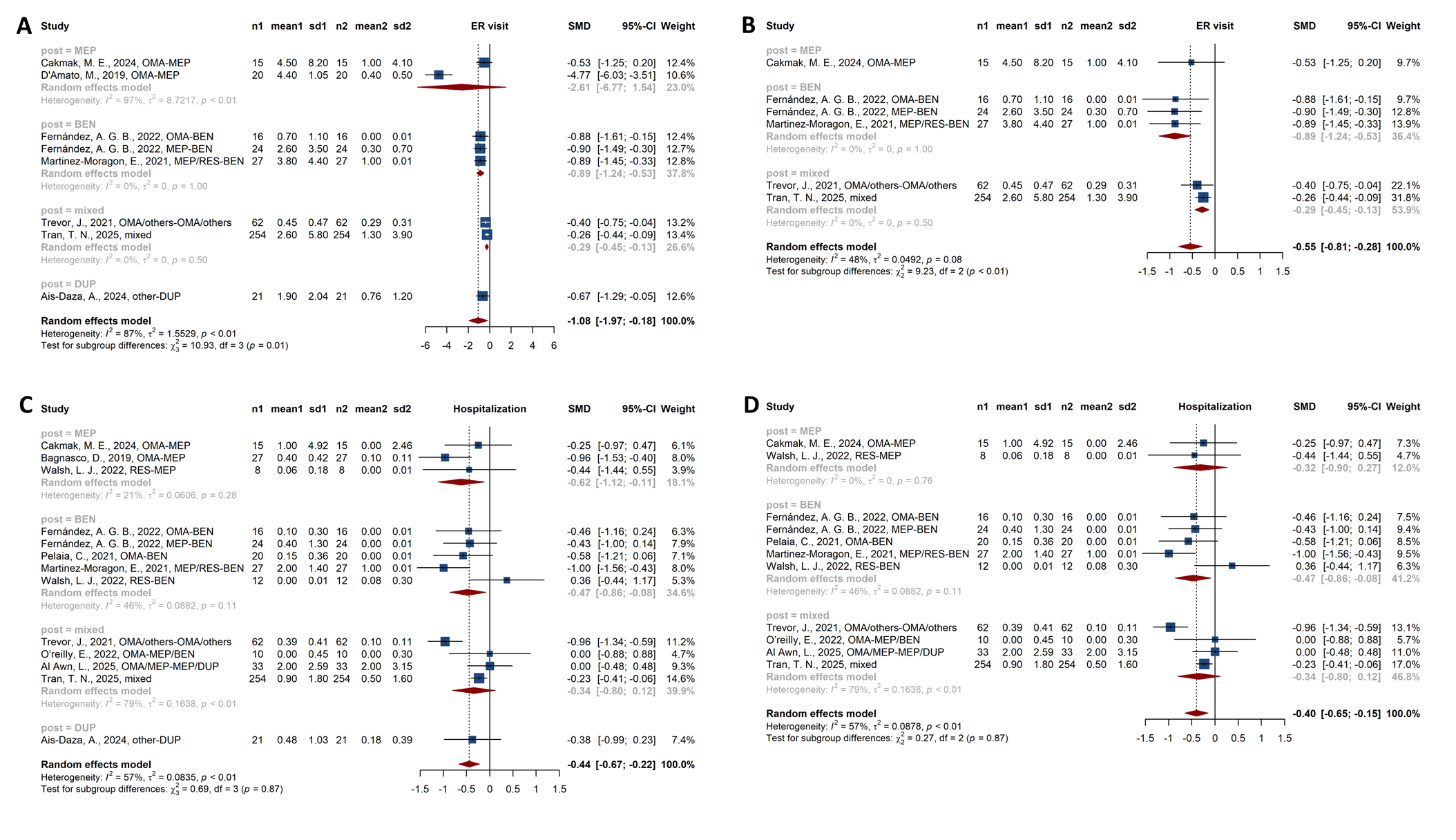


A.ER visit**; B. ER visit excluding publication type of conference abstract; C. Hospitalization; D. Hospitalizatoin excluding publication type of conference abstract**

**
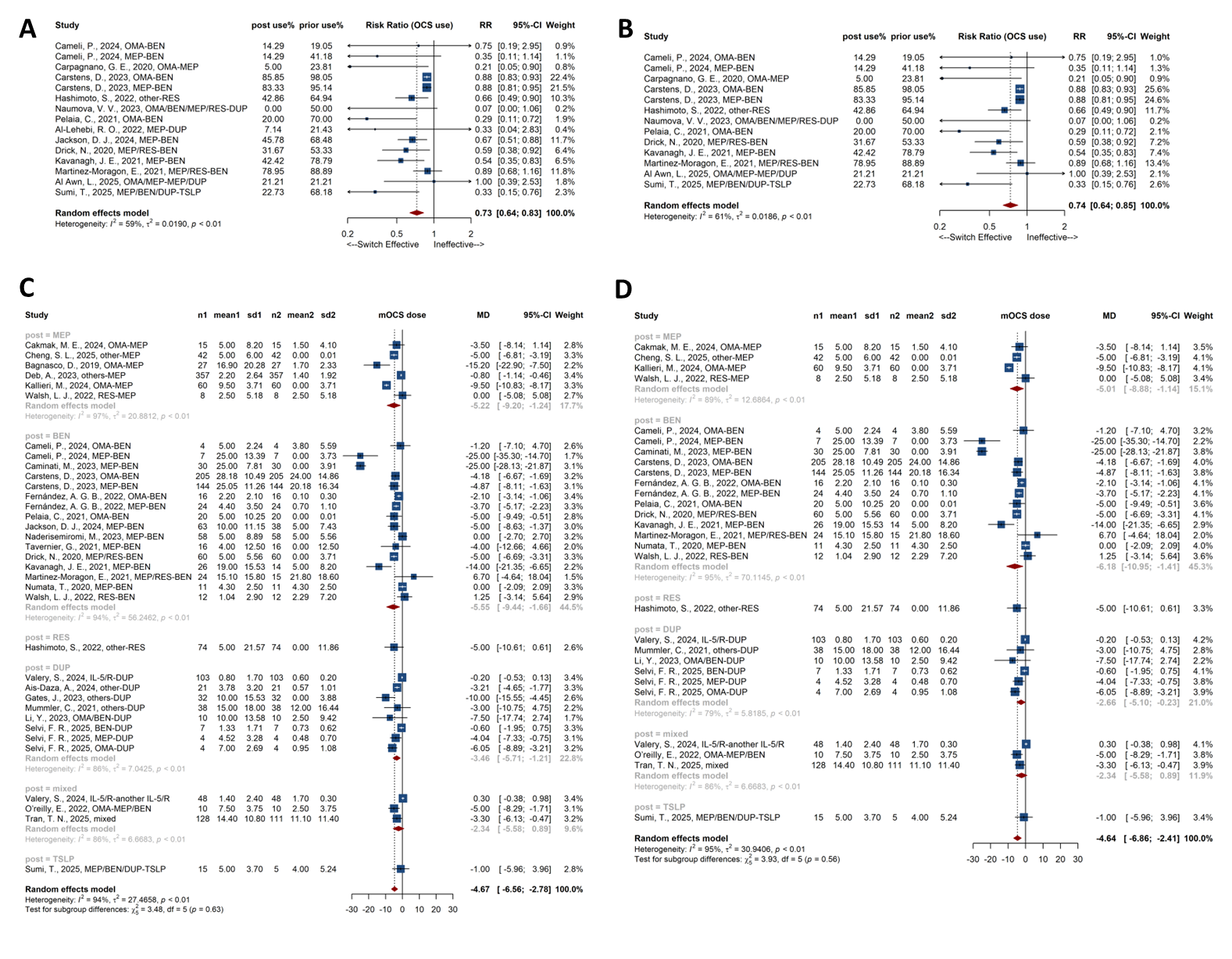
Supplemental Figure 4. OCS**

A.OCS use**; B.** OCS use **excluding publication type of conference abstract; C. maintenance OCS dose; D. maintenance OCS dose excluding publication type of conference abstract**


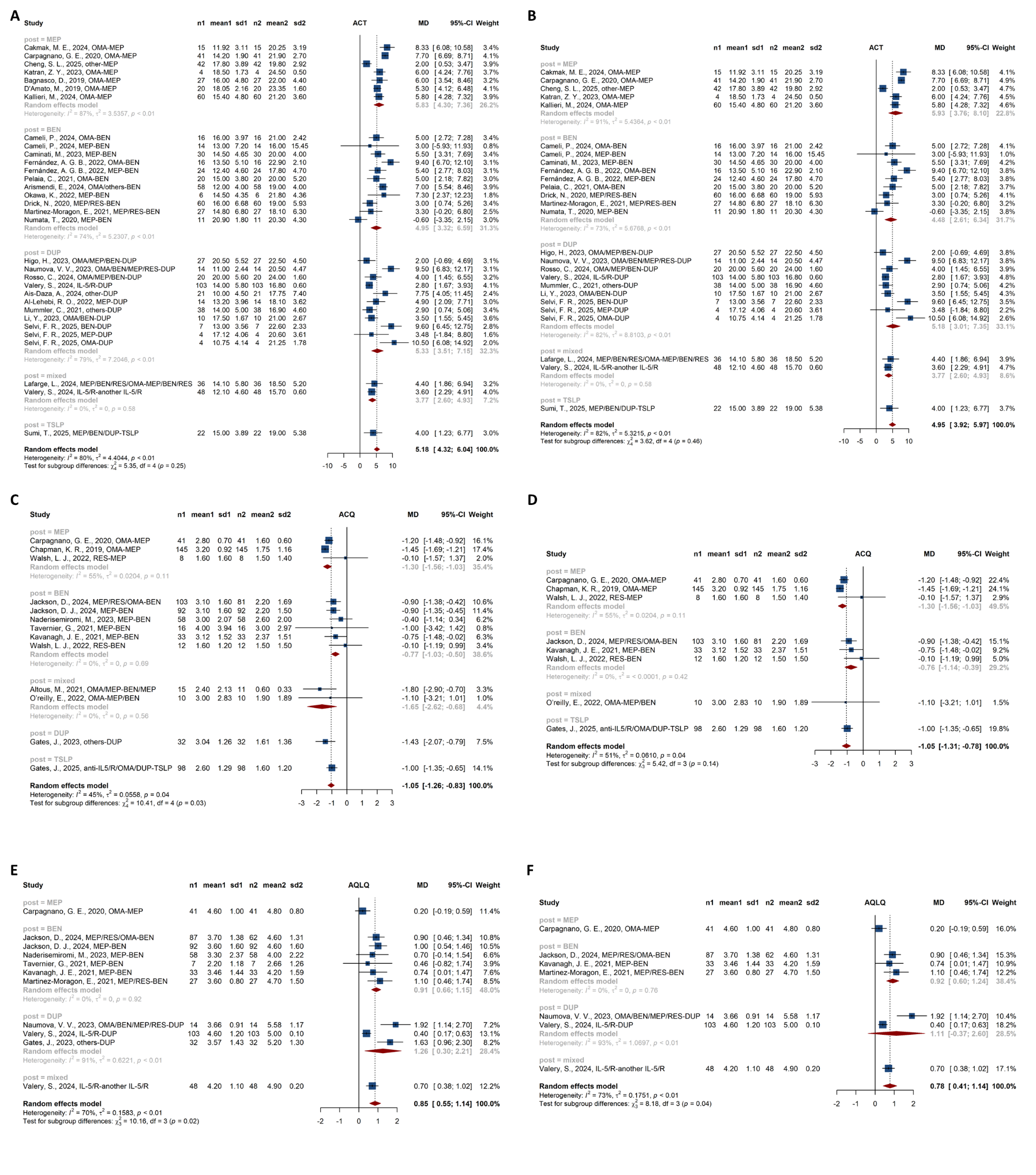
**Supplemental Figure 5. ACT, ACQ, AQLQ**

A. ACT score**; B.** ACT score **excluding conference abstract; C. mean ACQ score; D. mean ACQ score excluding conference abstract; E. mean AQLQ score; F. mean AQLQ score excluding conference abstract**

**
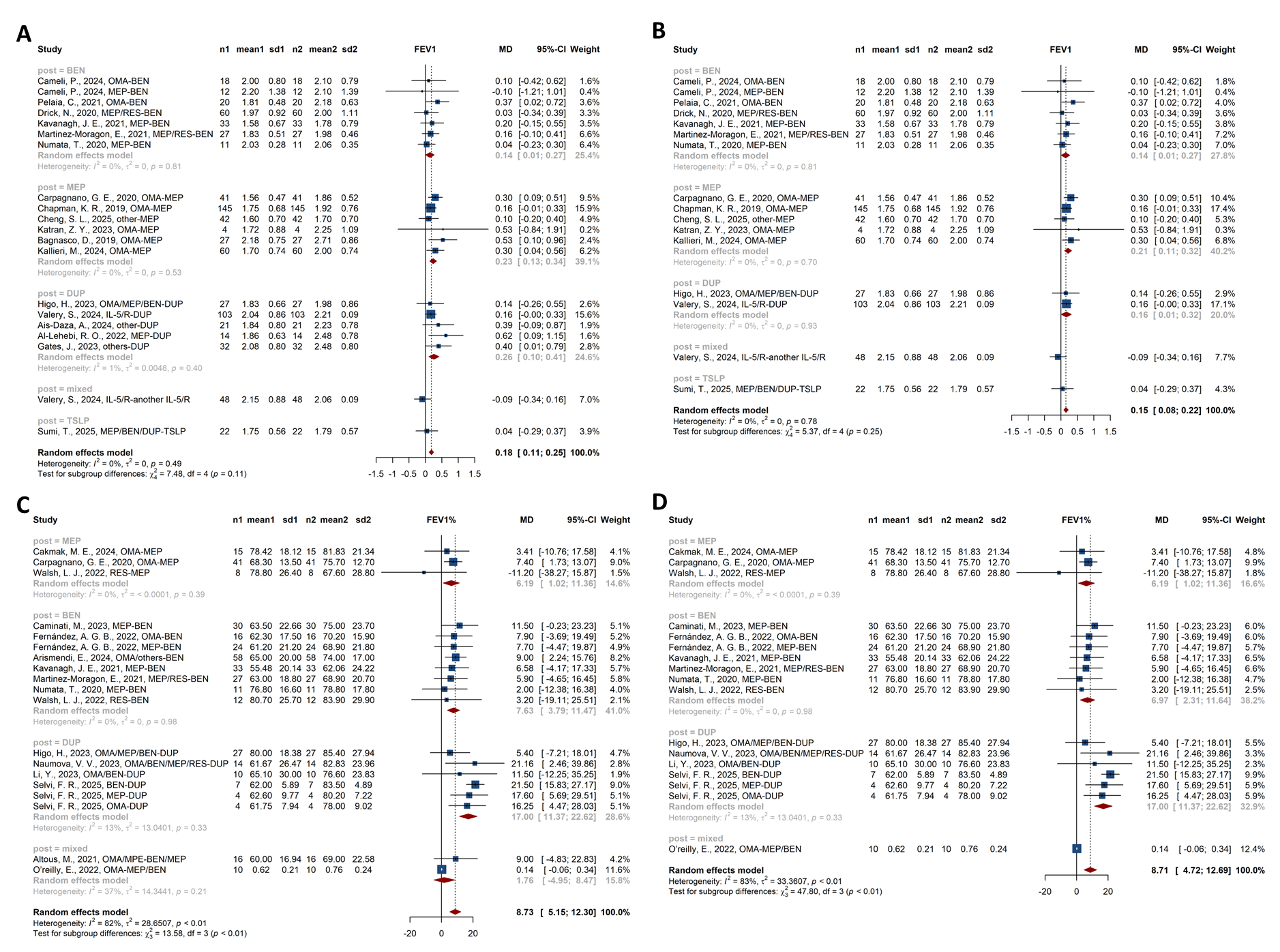
Supplemental Figure 6. FEV1**

A. Absolute FEV1; B. Absolute FEV1 excluding conference abstract; C. Percentage of FEV1 over predicted value (FEV1%); D. FEV1% excluding conference abstract

**
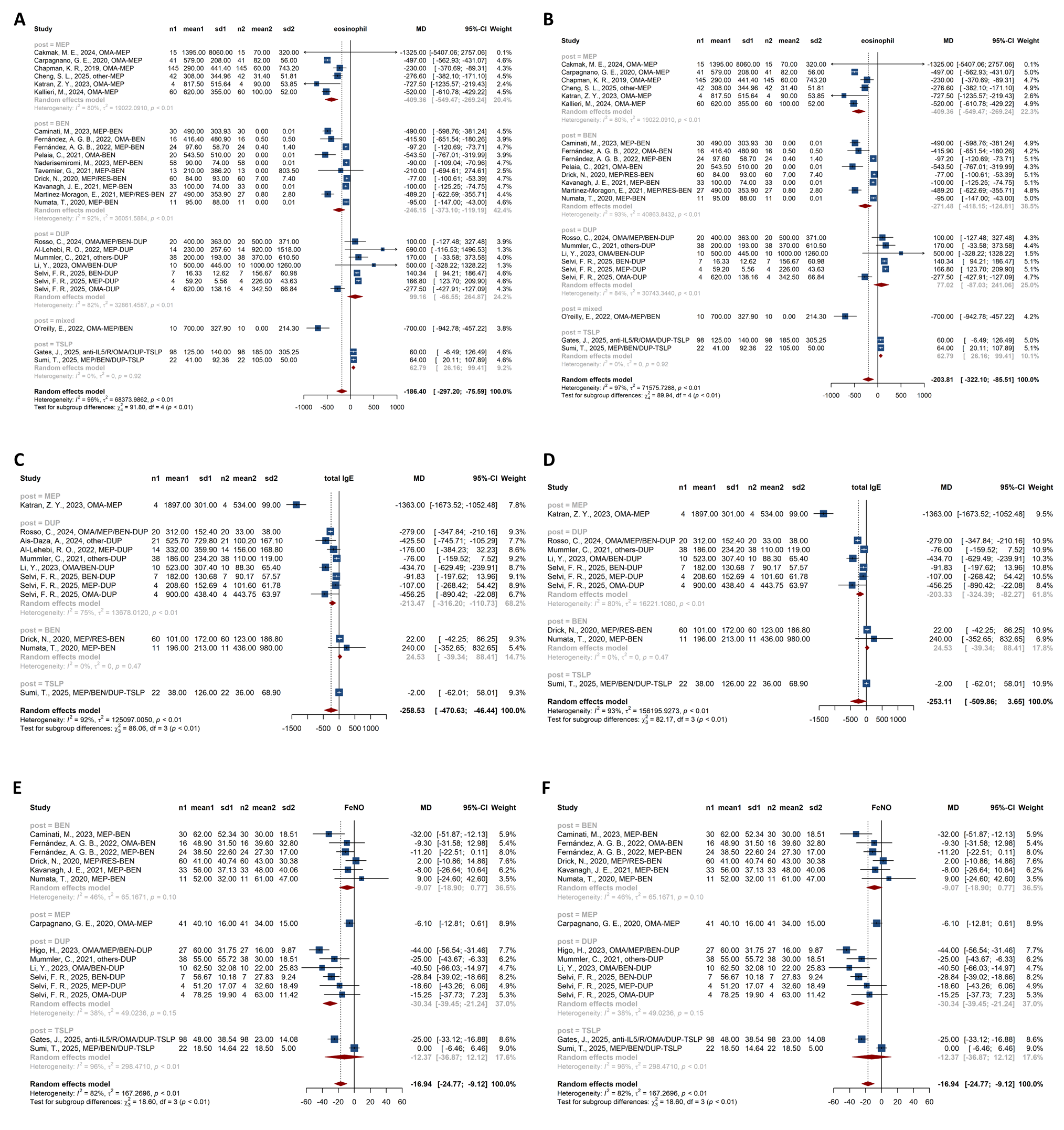
Supplemental Figure 7. T2-biomarkers**

A. Blood eosinophil count**; B.** Blood eosinophil count **excluding conference abstract; C. Total IgE level; D. Total IgE level excluding conference abstract; E. FeNO; F. FeNO excluding conference abstract**
